# Digital Biomarker–Based Studies: Scoping Review of Systematic Reviews

**DOI:** 10.2196/35722

**Published:** 2022-10-24

**Authors:** Hossein Motahari-Nezhad, Meriem Fgaier, Mohamed Mahdi Abid, Márta Péntek, László Gulácsi, Zsombor Zrubka

**Affiliations:** 1 Doctoral School of Business and Management, Corvinus University of Budapest Budapest Hungary; 2 Doctoral School of Applied Informatics and Applied Mathematics, Óbuda University Budapest Hungary; 3 Research Center of Epidemiology and Statistics Sorbonne Paris Cité, Paris University Paris France; 4 Health Economics Research Center, University Research and Innovation Center, Óbuda University Budapest Hungary; 5 Corvinus Institute for Advanced Studies, Corvinus University of Budapest Budapest Hungary

**Keywords:** scoping review, digital biomarkers, health, behavioral data, physiological data, digital health, remote monitoring, wearable, implantable, digestible, portable, sensor, digital health, mobile phone

## Abstract

**Background:**

Sensors and digital devices have revolutionized the measurement, collection, and storage of behavioral and physiological data, leading to the new term *digital biomarkers*.

**Objective:**

This study aimed to investigate the scope of clinical evidence covered by systematic reviews (SRs) of randomized controlled trials involving digital biomarkers.

**Methods:**

This scoping review was organized using the PRISMA-ScR (Preferred Reporting Items for Systematic Reviews and Meta-Analyses extension for Scoping Reviews) guidelines. With the search limited to English publications, full-text SRs of digital biomarkers included randomized controlled trials that involved a human population and reported changes in participants’ health status. PubMed and the Cochrane Library were searched with time frames limited to 2019 and 2020. The World Health Organization’s classification systems for diseases (International Classification of Diseases, Eleventh Revision), health interventions (International Classification of Health Interventions), and bodily functions (International Classification of Functioning, Disability, and Health [ICF]) were used to classify populations, interventions, and outcomes, respectively.

**Results:**

A total of 31 SRs met the inclusion criteria. The majority of SRs studied patients with circulatory system diseases (19/31, 61%) and respiratory system diseases (9/31, 29%). Most of the prevalent interventions focused on physical activity behavior (16/31, 52%) and conversion of cardiac rhythm (4/31, 13%). Looking after one’s health (physical activity; 15/31, 48%), walking (12/31, 39%), heart rhythm functions (8/31, 26%), and mortality (7/31, 23%) were the most commonly reported outcomes. In total, 16 physiological and behavioral data groups were identified using the ICF tool, such as looking after one’s health (physical activity; 14/31, 45%), walking (11/31, 36%), heart rhythm (7/31, 23%), and weight maintenance functions (7/31, 23%). Various digital devices were also studied to collect these data in the included reviews, such as smart glasses, smartwatches, smart bracelets, smart shoes, and smart socks for measuring heart functions, gait pattern functions, and temperature. A substantial number (24/31, 77%) of digital biomarkers were used as interventions. Moreover, wearables (22/31, 71%) were the most common types of digital devices. Position sensors (21/31, 68%) and heart rate sensors and pulse rate sensors (12/31, 39%) were the most prevalent types of sensors used to acquire behavioral and physiological data in the SRs.

**Conclusions:**

In recent years, the clinical evidence concerning digital biomarkers has been systematically reviewed in a wide range of study populations, interventions, digital devices, and sensor technologies, with the dominance of physical activity and cardiac monitors. We used the World Health Organization’s ICF tool for classifying behavioral and physiological data, which seemed to be an applicable tool to categorize the broad scope of digital biomarkers identified in this review. To understand the clinical value of digital biomarkers, the strength and quality of the evidence on their health consequences need to be systematically evaluated.

## Introduction

### Background

In health care systems, the use of digital devices has become an accelerating trend [1], and their application was sped up by the COVID-19 pandemic [2]. The emergence of new sensor-based devices and wearables has revolutionized measuring, collecting, and storing clinical data, which has definite consequences for clinical decision-making [3]. A new notion, digital biomarkers, has emerged in medicine: “objective, quantifiable, physiological and behavioral measures collected using digital devices that are portable, wearable, implantable or digestible” [4]. In addition to their clinical value, digital biomarkers enable new health care value chains [5]. According to published reports, the global digital biomarkers market size was valued at >US $727 million in 2019 and is predicted to grow at a compound annual growth rate of 40% to reach approximately US $10.38 billion by 2027 [6].

Digital biomarkers are measured across multiple layers of the hardware (eg, sensors) and software of medical devices that capture signals (behavioral and physiological data) from patients [7]. Digital biomarkers can increase diagnostic and therapeutic precision in the modern health care system by remotely and continuously measuring reliable clinical data and allowing continuous monitoring and evaluation [8,9]. Captured by wearable, implantable, and digestible devices and sensors, digital biomarkers can be used at home to provide clinical data, collecting data that is not possible in the clinical setting [10]. This information can improve physicians’ and patients’ decisions, personalize the treatment, and predict diseases’ current and future status [11]. Continuous evaluation allows personalized therapy [12]; for instance, continuous blood glucose monitoring by sensors in diabetes can be linked with patients’ physical activity and food intake data, which can tailor insulin dose adjustments and generate predictive alerts for critically low blood glucose levels [13]. In addition, digital biomarkers play an essential role in the recognition of disease-related symptoms [14], are commonly used in clinical trials to evaluate different therapies [15], and offer better treatment, especially when combined with other interventions [16]. Overall, digital biomarkers play a significant role in precision medicine [17], can reduce clinical mistakes, improve the accuracy of diagnostic methods, and support personalized clinical decisions [18].

Several systematic reviews (SRs) have been published on digital biomarkers. However, most of them focused on a specific technology or disease area; for instance, studies reviewed the health impacts of wearable activity trackers on a general population [19] and in patients with Parkinson disease [20], to name a few that covered specific technologies in specific disease areas [21-23]. Scoping reviews aim to capture the main concepts of a research area and the available primary sources and categories of evidence in a formal, rigorous, and transparent manner [24]. Digital biomarkers cover various clinical areas such as Fitbit devices, activity trackers, and implantable cardiac defibrillators. The potential value of digital biomarkers in effective, technologically enhanced, safe, and user-centered care pathways [25,26] has been suggested by a plethora of published SRs on their clinical benefits in various clinical areas. However, no scoping review has covered all digital biomarkers in a single, complete study. An overview of the scope of clinical evidence can highlight clinical areas where the evidence supports the integration of digital biomarkers into health systems and areas with gaps in the evidence synthesis. Therefore, a scoping review of SRs on digital biomarkers may help readers grasp the breadth of the accumulated clinical evidence in the field. As the Cochrane Handbook states, reviews of reviews address the need for broad evidence synthesis by covering multiple interventions for the same condition as well as numerous reviews of the same intervention for different disease areas [27].

### Objectives

Given the rapid accumulation of clinical evidence partly driven by the COVID-19 pandemic and the new European Medical Device Regulation that took effect in May 2021, this scoping review includes SRs published in 2019-2020 to determine in which clinical domains digital biomarkers and sensors were making progress before the new regulation took effect. Specifically, this scoping review aimed to explore the following:

1. The characteristics of SRs of digital biomarkers in terms of populations, interventions, and outcomes.
2. The characteristics of digital biomarkers in terms of behavioral and physiological data types, the digital devices and sensors used, and their role in the treatment pathway in the SRs.

The purpose of this scoping review was to categorize the building blocks of the research questions, not to synthesize or evaluate the quality of clinical evidence on digital biomarkers; this will be addressed in a separate SR of SRs of digital biomarker–based interventions, which will assess the methodological quality and quality of evidence for digital biomarkers in meta-analyses using A Measurement Tool to Assess Systematic Reviews-2 and Grading of Recommendations Assessment, Development, and Evaluation, respectively [28].

## Methods

We followed the PRISMA-ScR (Preferred Reporting Items for Systematic Reviews and Meta-Analyses extension for Scoping Reviews) guidelines [29].

### Eligibility Criteria

According to the definition [4], digital biomarkers are behavioral and physiological data such as heart rate, physical activity, and step counts collected using digital devices such as smartwatches [30]. Accordingly, in this study, we identified digital biomarkers as behavioral and physiological data that were measured using digital devices. Digital technologies that do not objectively quantify physiological or behavioral data were excluded from this study. To focus on the evidence relevant to clinical care, we included SRs that involved randomized controlled trials (RCTs), indicating that the review synthesized causal evidence concerning health outcomes [31]. Studies that did not report changes in participants’ health status were excluded. In addition, SRs containing only observational studies were inappropriate for this study. Following the general definition of SRs [32], we included studies that used a systematic search strategy in electronic databases and had a predefined and clear research question, inclusion and exclusion criteria, screening, and data analysis and synthesis methods. Reviews lacking the critical appraisal of the included studies were considered SRs if other criteria were met [32]. We did not restrict our scoping review to a specific population. Human studies in any clinical setting and any age group or sex were eligible for this study. We considered all interventions that intentionally or unintentionally influence the health status of participants and involve the use of at least one digital biomarker for any purpose related to diagnosing patients, monitoring outcomes, or affecting the delivery of the therapeutic intervention or for prognostic purposes. We did not limit the scoping review to any specific type of comparator group. Full-text English-language SRs that considered any kind of health outcome (eg, change in the health status of individuals or a population due to an intervention) [31] were eligible for this scoping review.

### Exclusion Criteria

Studies were excluded if (1) they were not SRs; (2) all included studies in the SR were not RCTs; (3) they were not human studies; (4) they did not use at least one digital biomarker to diagnose patients, monitor outcomes, or influence the delivery of the therapeutic intervention or for prognostic purposes; (5) they did not use at least a wearable, implantable, portable, or digestible device to measure behavioral or physiological data; (6) they did not report health outcomes (ie, they did not report a change in population health status due to the use of an intervention); (7) they were not published in full text written in English; and (8) they had not been published in 2019 or 2020.

### Search Strategy

A comprehensive strategy for searching published SRs was established, including the following steps. First, the PubMed electronic database was searched using keywords related to the definition of digital biomarkers in the title or abstract, as well as applicable Medical Subject Headings terms [4], combined with the National Library of Medicine’s filter for SRs [33]. Second, the Cochrane Library database of SRs was also searched using keywords related to digital biomarkers. The search was limited to studies published in 2019 or 2020. Finally, during the review process, an additional investigation was conducted into the reference lists of identified studies.

We used the following digital biomarker–related search terms: “digital biomarker” OR “digital biomarkers” OR “implantable” OR “implantables” OR “wearable” OR “wearables” OR “portable” OR “portables” OR “digestible” OR “digestibles” [28] (refer to Multimedia Appendix 1 for details of the search strategy).

### Screening and Selection of Studies

Microsoft Excel was used to manage articles and remove duplicate references according to their digital object identifier numbers. Two independent reviewers selected the reviews in 2 phases as follows:

1. Titles and abstracts of retrieved records were screened to identify relevant studies based on the following 2 inclusion criteria: Is this an SR study (yes or uncertain, no)? and Is it a digital biomarker–based study (yes or uncertain, no)? The studies for which the answer to both questions was yes or uncertain were considered eligible for the next step.
2. The full texts of articles that met the criteria for title and abstract were assessed based on the following binary (yes or no) factors: whether the study was published in 2019 or 2020; whether it was written in English; whether it was a human study; whether the study included only RCTs; whether health outcomes were reported; and whether there was at least one digital biomarker in the study for diagnosing patients, monitoring outcomes, and influencing therapeutic intervention or use of a wearable, implantable, portable, or digestible device for prognostic purposes. Filtering the answers of all questions to *yes* identified all eligible studies. At each screening stage, disagreements were discussed between the two reviewers and resolved by consensus (HM-N, MMA, and MF). At each screening stage, the interrater agreement between the reviewers was calculated using the Cohen κ statistic using Microsoft Excel. The substantial agreement rate was considered to be κ>0.6 [34]. In case of low agreement (κ<0.6), the reviewers were retrained before entering the full-text phase.

### Data Charting

#### Overview

Two review authors independently extracted data from the included reviews and discussed their findings to ensure consistency. All entries were cross-checked. We used charting data forms to extract data. Where possible, the data were copied and pasted directly from the text to avoid misinterpretation. Regarding the agreement rate, Cohen κ [34] was calculated using Microsoft Excel. In terms of countries, populations; interventions; outcomes; behavioral and physiological data; role of digital biomarkers; type of sensor technology; and descriptive statistics, including frequency and percentage, were calculated using Stata statistical software (version 16.0; StataCorp LLC) and Microsoft Excel. Regardless of country, type of digital device, and role of digital biomarkers, the total frequency and percentage of the other variables mentioned do not correspond to the total number of SRs included (31% and 100%, respectively) because an SR may have more than one category of these variables, as shown in the reported results. R statistical software (version 4.1.3; The R Foundation for Statistical Computing) was used to visualize the graphs. We did not assess the quality of the included reviews because this is not essential in scoping reviews [29].

#### The Characteristics of SRs

We used the classification systems developed by the World Health Organization (WHO) to characterize populations, interventions, and outcomes. The International Classification of Diseases, Eleventh Revision (ICD-11), is the latest disease classification system [35]. The International Classification of Health Interventions (ICHI) is a tool proposed for statistical classification, documenting, and analyzing of health interventions [36]. The ICHI encompasses diagnostic, medical, surgical, mental health, primary care, and allied health services; practical support; rehabilitation; traditional medicine; and public health initiatives offered by a wide range of professionals throughout the spectrum of health care systems. The International Classification of Functioning, Impairment, and Health (ICF) is a framework for assessing health and disability at the individual and population levels [37]. The populations studied in the included SRs were categorized using the ICD-11. In addition, the populations’ age range (children, adults, older adults, other, or unspecified) and sex (male, female, or both male and female) were extracted. The interventions and outcomes were also grouped using the ICHI and ICF instruments, respectively. The ICF instrument was also used to classify physiological and behavioral data. In addition, the comparison types were collected from the included SRs. We also extracted the number of included RCTs in the SRs.

#### The Characteristics of Digital Biomarkers

The characteristics of digital biomarkers were recorded, including their role in the SR (intervention, measure of outcome, diagnostic tool, prognostic tool, or other), the type of physiological and behavioral data gathered by digital devices using the ICF tool [37], the type of digital device (implantable, portable, wearable, or digestible), and the type of applied sensor technology (biosensor, chemical sensor, flow sensor, fingerprint sensor, force sensor, heart rate sensor or pulse rate sensor, humidity sensor, hour monitor sensor, infrared sensor, image sensor, level sensor, muscle sensor, position sensor, pressure sensor, thermistor sensor, or temperature sensor) [38].

We identified sensors such as heart rate sensors and pulse rate sensors if they were involved in sensing cardiac rhythm and function (heart rate sensors) and blood pressure (pulse rate sensors). By contrast, position sensors were assigned to those reviews assessing physical activities, walking, running, or gait functions. In addition, sensors related to smoking behavior were grouped into flow sensors. The sensors that monitored body temperature were categorized as temperature sensors.

### Evidence Synthesis

This scoping review used descriptive-analytical methods, including frequency, percentage, and data charting using Stata statistical software (version 16.0). The screening process was evaluated by calculating Cohen κ between the independent pairs of reviewers. The graphs were designed using R statistical software (version 4.1.3).

## Results

### Screening and Selection of Studies

**Figure 1:**
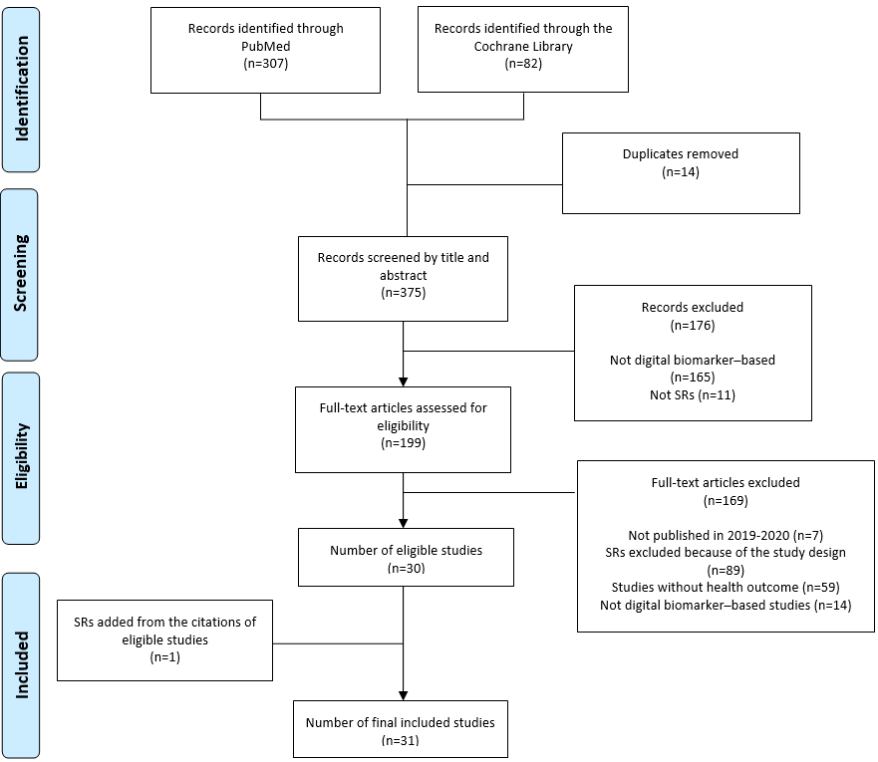
PRISMA (Preferred Reporting Items for Systematic Reviews and Meta-Analyses) diagram of the selection and screening process. SR: systematic review.

From the computerized searches, 389 records were identified: 307 (78.9%) and 82 (21.1%) records in the PubMed and Cochrane Library databases, respectively. After removing duplicates, of the 389 records, 375 (96.4%) were screened for titles and abstracts. During title and abstract screening, there were 87 disagreements between the reviewers (Cohen κ=0.54). Therefore, the reviewers were retrained to reach a higher level of agreement. Consequently, they entered the discussion phase to resolve the discrepancies. In the screening phases of the titles and abstracts, 94% (82/87) of the disagreements were associated with *digital biomarker* and 6% (5/87) with *systematic review*. Of the 375 papers screened for titles and abstracts, 199 (53.1%) full-text papers were selected for the evaluation of eligibility. After resolving 42 disputes in the study selection phase (Cohen κ=0.76; n=17, 40%, disagreements on *health outcome*; n=14, 33%, disagreements on *digital biomarker–based studies*; n=9, 21%, disagreements about *RCTs*; and n=2, 5%, disagreements regarding *published in 2019-2020*), 44.7% (89/199) of the SRs were excluded at the full-text screening phase because of the study design (Multimedia Appendix 2). Of the 110 remaining SRs, 30 (27.3%) matched the inclusion criteria. After checking the reference lists of the qualifying SRs, one more record was included, bringing the total number of SRs that fit the inclusion criteria to 31. The PRISMA (Preferred Reporting Items for Systematic Reviews and Meta-Analyses) flowchart is shown in Figure 1. The characteristics of the studies are summarized in Multimedia Appendix 3 [39-69]. (Refer to Multimedia Appendix 4 for the list of excluded studies and the reasons for exclusion).

### Characteristics of the Included SRs

The included SRs were published by authors from 14 different countries, most of them from Australia (6/31, 19%) [39-44], followed by Canada (4/31, 13%) [45-48]; the United States (4/31, 13%) [49-52]; the United Kingdom (3/31, 10%) [53-55]; Hong Kong (3/31, 10%) [56-58]; Taiwan (2/31, 7%) [59,60]; and Belgium (2/31, 7%) [61,62]. The remaining 7 studies were published by Chinese [63], French [64], Japanese [65], Portuguese [66], Italian [67], Dutch [68], and Danish [69] researchers.

#### Populations

Participants’ disease areas covered 13 ICD-11 chapters (Table 1). The majority of SRs studied participants with circulatory system diseases (19/31, 61%) [40,41,45,46,49,51,53-55, 57,60,62-69], followed by respiratory system diseases (9/31, 29%) [43,46,50,57,61-65]; endocrine, nutritional, or metabolic diseases (7/31, 23%) [40,45,50,57,62,64,65]; sleep-wake disorders (4/31, 13%) [50,57,63,64]; diseases of the nervous system (4/31, 13%) [40,57,63,64]; neoplasms (3/31, 10%) [40,52,64]; factors influencing health status or contact with health services (3/31, 10%) [40,56,60]; mental, behavioral, or neurodevelopmental disorders (2/31, 7%) [63,64]; diseases of the genitourinary system (2/31, 7%) [58,64]; and diseases of the musculoskeletal system or connective tissue (2/31, 7%) [40,64]. Moreover, the study by Lu et al [63] included patients with visual system diseases (1/31, 3%). Injury, poisoning, or certain other consequences of external causes as well as patients with skin diseases were the eligible included populations in another SR [64]. In 19% (6/31) of the studies, the included populations were only nonclinical and general participants [39,42,44,47,48,59]. In spite of comprising patients with the aforementioned specific clinical conditions, some SRs also included general populations without an applicable ICD-11 category, such as employees (3/31, 10%) [40,56,61]; students (3/31, 10%) [40,46,50]; healthy participants (2/31, 7%) [40,53]; and office workers (1/31, 3%) [40].

**Table 1:** Disease areas were identified using the International Classification of Diseases, Eleventh Revision, tool (N=31).

Populations	Values, n (%)
Diseases of the circulatory system	19 (61)
Diseases of the respiratory system	9 (29)
Endocrine, nutritional, or metabolic diseases	7 (23)
Diseases of the nervous system	4 (13)
Sleep-wake disorders	4 (13)
Neoplasms	3 (10)
Factors influencing health status	3 (10)
Mental, behavioral, or neurodevelopmental disorders	2 (6)
Diseases of the genitourinary system	2 (6)
Diseases of the musculoskeletal system	2 (6)
Diseases of the visual system	1 (3)
Diseases of the skin	1 (3)
Consequences of external causes	1 (3)

#### Interventions

According to the ICHI classification (Table 2), a high proportion of the interventions focused on physical activity behavior (16/31, 52%) [39-46,48,52,55-57,61,62,68] and conversion of cardiac rhythm (4/31, 13%) [49,53,66,67]. Percutaneous transluminal destruction of the arrhythmia circuit was covered by 7% (2/31) of the SRs [51,54]. Other SRs concerned assessment of weight maintenance functions (2/31, 7%) [50,59]; cardiac electrophysiological monitoring (1/31, 3%) [60]; assisting or leading exercise for functions of the cardiovascular system (1/31, 3%) [69]; assisting or leading exercise for functions related to pregnancy (1/31, 3%) [58]; blood pressure functions (1/31, 3%) [50]; noneconomic incentives to encourage improved physical activity (1/31, 3%) [65]; and economic incentives to encourage improved physical activity (1/31, 3%) [47]. The study by Jo et al [50] included 4 different types of interventions, but only 2 of these interventions could be categorized by the ICHI instrument: weight maintenance function and blood pressure function. The other 2 intervention types—blood cholesterol monitoring and wearable blood glucose monitoring systems—could not be categorized with the ICHI tool. Besides, 7% (2/31) of the SRs [63,64] did not assign an intervention category because they did not define a specific type of intervention; therefore, assignment to a specific intervention was not possible with the ICHI tool.

**Table 2:** Categorization of interventions using the International Classification of Health Interventions tool (N=31).

Interventions	Values, n (%)
Assessment of physical activity behaviors	16 (52)
Conversion of cardiac rhythm	4 (13)
Percutaneous transluminal destruction of arrhythmia circuit	2 (6)
Assessment of weight maintenance functions	2 (6)
Cardiac electrophysiological monitoring	1 (3)
Assisting or leading exercise for functions of the cardiovascular system	1 (3)
Assisting or leading exercise for functions related to pregnancy	1 (3)
Blood pressure function	1 (3)
Noneconomic incentives to encourage improved physical activity	1 (3)
Economic incentives to encourage improved physical activity	1 (3)

#### Outcomes

The reported outcomes fell into 13 unique categories of the ICF. Looking after one’s health (physical activity; 15/31, 48%) [39-41,43-48,52,55-57,64,68]; walking (12/31, 39%) [39-43,46,52,56,57,61,62,68]; heart rhythm (8/31, 26%) [49,51,53,54,60,66,67,69]; demographic change (mortality; 7/31, 23%) [49,51,53,54,66,67,69]; and weight maintenance functions (7/31, 23%) [42,46-48,50,55,59] were the most commonly reported outcomes in the studies. Blood pressure functions (3/31, 10%) [50,55,64] and heart functions (3/31, 10%) [54,63,69] were the primary types of outcomes in 3 distinct SRs each. In addition, the following classifications were each assigned to one review: aerobic capacity (1/31, 3%) [41]; functions related to pregnancy (1/31, 3%) [58]; sleep functions (1/31, 3%) [64]; and heart rate (1/31, 3%) [64]. Refer to Table 3 for further details.

**Table 3:** Categorization of outcomes using the International Classification of Functioning, Disability, and Health tool (N=31).

Outcomes	Values, n (%)
Looking after one’s health	15 (48)
Walking	12 (39)
Heart rhythm	8 (26)
Demographic change (mortality)	7 (23)
Weight maintenance functions	7 (23)
Blood pressure functions	3 (10)
Heart functions	3 (10)
Hematological system functions	2 (6)
Exercise tolerance functions	2 (6)
Aerobic capacity	1 (3)
Functions related to pregnancy	1 (3)
Sleep functions	1 (3)
Heart rate	1 (3)

### Characteristics of Digital Biomarkers

The behavioral and physiological data characteristics, digital devices, and sensors are summarized in Multimedia Appendix 5 [39-69].

#### Behavioral and Physiological Data and Digital Devices

Digital biomarkers were extracted from the included SRs. In total, 16 physiological and behavioral data groups were identified using the ICF tool, such as looking after one’s health (physical activity; 14/31, 45%) [39,40,43-48,52,55-57,64,68]; walking (11/31, 36%) [39-43,52,56,57,61,62,68]; heart rhythm (7/31, 23%) [49,51,53,54,66,67,69]; and weight maintenance functions (7/31, 23%) [42,46-48,50,55,59]. The other identified data can be found in Multimedia Appendices 5 and 6. Besides, various digital devices were also used to collect these data when assessing other interventions; for example, an implantable cardiac defibrillator to gather heart function data [60]; a Fitbit device for capturing running activity [46]; Yorbody and AiperMotion for capturing physical activity [45]; and smart glasses, smartwatches, smart bracelets, smart shoes, and smart socks for capturing data related to heart function, gait pattern function, and temperature [63]. For more information, refer to Multimedia Appendix 5.

#### Role of Digital Biomarkers in Clinical Care

A substantial number of digital biomarkers were used as interventions in the SRs (24/31, 77%) [39-46,48-50,52,53,55-62,65-67]. By contrast, digital biomarkers were used to measure outcomes in 10% (3/31) of the studies [47,64,68]. In addition, in the review by Lu et al [63], digital biomarkers were used as intervention as well as outcome measurement and diagnostic tools. The remaining studies (3/31, 10%) [51,54,69] did not use digital biomarkers as intervention or diagnostic tools, as prognostic tools, or to measure outcomes; we categorized the role of digital biomarkers as *other*. In these studies, the included populations were patients with digital biomarkers (implantable cardiac defibrillators).

#### Types of Sensor Technologies

Wearables (22/31, 71%) were the most common types of digital devices [39-48,50,52,55,57-59,61-65,68], followed by implantable devices (8/31, 26%) [49,51,53,54,60,66,67,69]. The study by Liu et al [56] included both wearable and portable digital devices. Position sensors (21/31, 68%) [39-48,50,52,55-59,61-63,68,69] and heart rate sensors and pulse rate sensors (12/31, 39%) [49-51,53-55,60,63,64,66,67,69] were identified as the most prevalent types of sensors used to acquire behavioral and physiological data in the reviews. Flow (1/31, 3%) [64] and temperature (1/31, 3%) [63] sensors were used in 1 review each. Multimedia Appendix 5 shows the included studies and the role and types of digital devices, sensors, and physiological and behavioral data.

## Discussion

### Principal Findings

This scoping review of SRs of digital biomarkers published in 2019-2020 aimed to determine the scope of the literature in terms of populations, interventions, outcomes, technologies used, behavioral and physiological data, device types, and sensors. The search yielded 31 SRs that met the inclusion criteria and were published primarily by Australian, Canadian, American, and British researchers. The results showed that most of the populations studied were patients with circulatory, respiratory, endocrine, nutritional, or metabolic diseases. Intervention types were also predominantly used to assess physical activity behaviors and cardiac rhythm conversion. Wearables were the most common types of digital devices, mainly as interventions in the form of position and heart rate sensors.

There are numerous scoping reviews in this area, divided into 2 categories. First, scoping reviews focus on a specific type of digital device; for example, the study by Brognara et al [70] examined wearable sensors for assessing gait and postural alterations in patients with diabetes. Another scoping review highlighted the scope of wearable technologies in field hockey competitions [71]. Second, some scoping reviews considered only a specific behavioral or physiological data type; for example, the scoping review by Youn et al [21] examined digital biomarkers for neuromuscular disorders. Another study also reported the capabilities of artificial intelligence–aided digital biomarkers to aid in the early detection of dementia [72]. Therefore, we did not restrict the study to a particular digital device or behavioral or physiological data type to establish comprehensive results on digital biomarkers.

### Populations, Interventions, and Outcomes

According to the findings, the populations, interventions, and outcomes studied in the SRs predominantly fall into 2 groups: physical activity and cardiovascular diseases. Although only 13 chapters of the ICD-11 and 10 categories of the ICHI were included in the SRs, because of the rapid pace of developments in the field and the fact that digital health and digital biomarkers are in transition [73,74], it is expected that the number of studies in other categories will increase. In addition, new devices, digital biomarkers, and sensors are expected to be introduced in health care systems and various disease areas because of the new advancements [75].

SRs are essential to evidence-based practice and health care decision-making [27,76]. According to the Cochrane Handbook, formulating a research topic based on population, intervention, comparison, and outcomes is one of the most significant requirements for SRs [27]. Although 61% (19/31) of the included SRs explicitly described a particular group with a clinical condition such as chronic obstructive pulmonary disease [43], others (6/31, 19%) included populations without clinical disorders [39,42,44,47,48,59]. By contrast, 19% (6/31) of the studies did not restrict their targeted population to a particular therapeutic area; rather, they encompassed diverse disease areas [46,50,57,62-64]. As evidenced by the findings, several reviews included general populations (patients with nonclinical conditions) to whom ICD-11 codes could not be assigned. As the use of wearable devices and sensors is spreading to broad populations such as students, employees, and office workers, and certain studies (RCTs) have included these populations, ICD-11 coding should include these populations. This issue also applies to interventions. Some (2/31, 6%) of the SRs did not clearly define the type of intervention in their study. Wearable health devices in health care settings, as well as pharmacologic and nonpharmacologic interventions, are not specific enough to be categorized using the ICHI tool. Therefore, researchers in this area are advised to consider this issue when formulating their research questions. In addition, 2 types of interventions (blood cholesterol monitoring and wearable blood glucose monitoring systems) could not be categorized with the ICHI tool, which should be considered by the developers of the tool.

Although some (11/31, 35%) of the studies evaluated the impact of a single kind of digital technology on the population, such as a Fitbit device [46], an implanted cardiac defibrillator [53], or a pedometer [61], others (20/31, 65%) included a variety of technologies [40,63-65]. In this context, the Cochrane Handbook suggests that reviews of reviews may be one way to address the need for breadth in evidence synthesis because they may combine multiple reviews of different interventions for the same condition or numerous reviews of the same intervention for different types of participants [27]. This is particularly true for the digital biomarker literature because this area encompasses a wide variety of populations and therapies, as demonstrated by this study’s results.

### Digital Devices, Physiological and Behavioral Data, and Sensors

Although numerous studies have shown that the accuracy of digital devices in measuring behavioral and physiological data may vary [77-80], most (20/31, 65%) of the included studies used different digital devices to synthesize qualitative or quantitative findings, which can be considered a gap in the literature on digital biomarkers; for example, the SR by Hannan et al [41] included various wearable digital devices (Garmin Forerunner, Fitbit Charge, My Wellness Key accelerometer, Yamax Digiwalker pedometer, Gex vital signs sensor, Nokia smartphone, and SenseWear Mini Armband) as interventions to quantitatively summarize the evidence for cardiac rehabilitation. By contrast, another study used only a Fitbit device to generate a meta-analysis for physical activity [46]. Despite the growing acceptance of wearables, the widespread adoption of wearables in clinical practice is still hampered by several barriers, including concerns about device accuracy and cost. To overcome these barriers, multiple stakeholders must collaborate in developing comprehensive assessment frameworks, clinical trials, and medical education programs. However, companies developing digital health technologies should consider the importance of evidence generation and validation for digital devices [7], considering that verification and validation of digital biomarkers require a multidisciplinary approach that includes engineering, data science, health information technology, and clinical research [8].

As shown in Multimedia Appendix 5, various digital devices are used to collect the same behavioral and physiological data; for example, an implantable cardiac defibrillator, iPhone-based rhythm monitoring device [60], and Cardio First Angel [81] to capture cardiac functions. Health economics research should evaluate the cost-effectiveness of each device in collecting behavioral and physiological data to determine the most cost-effective digital device for collecting specific data; for example, a study examined the potential cost-effectiveness of a wearable cardioverter defibrillator for patients with implantable cardiac defibrillator explant in a high-income Chinese city. It concluded that the cost-effectiveness of the wearable cardioverter defibrillator was highly dependent on the daily cost of the device in China [81]. Another study that examined the cost-effectiveness of portable devices for stroke diagnosis found no evidence of the cost-effectiveness under consideration for stroke diagnosis [82]. Another question that might arise from our results is the clinical effectiveness as well as cost-effectiveness of different digital devices; for example, portable devices (tablet computers) and wearables (Jawbone UP24) were used to measure physical activity [56], or implantable cardiac monitors and portable devices (Holter electrocardiogram) were applied to record cardiac parameters [83]. Still, the question of comparative assessment of clinical effectiveness and cost-effectiveness between wearables and portables has remained unanswered. Digital health is undergoing rapid change, and new digital devices are being integrated into health care systems to facilitate it [84]. However, research shows that in medicine, more is not necessarily better [85], and rigorous evaluation of such therapies will become increasingly important in the future [84]. As a result, the cost-effectiveness of similar digital technologies could help clinicians and policy makers improve health care quality and reduce clinical costs.

We also used a simple search syntax derived from the definition of digital biomarkers. Still, the precision and specificity of this search formula to find all relevant studies have not been determined. Hence, the question regarding the development of a comprehensive and authoritative search formula with high precision and specificity has remained unanswered and is beyond the scope of this research. One of the significant challenges in digital health or biomarkers is the lack of a standard definition and mechanism for researchers to use when formulating a search syntax for reviews [82]. Standardizing definition terms is paramount to enhancing information retrieval and evidence synthesis. Accordingly, the quality of evidence synthesis of digital biomarkers may be compromised by publication bias, resulting in lower certainty of the evidence [83]. Digital biomarkers are an emerging field in flux and encompass various technologies. As we explained in the Methods section, digital biomarkers, by definition, are physiological and behavioral data collected using digital devices, including wearables as well as implantable, portable, and digestible devices. As can be inferred from the definition, there are 2 concepts to consider with digital biomarkers: behavioral and physiological data and digital devices that capture these data. However, no SR of portable digital devices that satisfied the inclusion requirements for this scoping study was discovered. This issue may result from the search formula obtained from the definition or a gap in the literature.

The varieties of digital biomarkers used in different health domains require specialized definitions, standards, and methodologies for achieving integration [84,85]. We used the ICF tool in this study to identify and classify behavioral and physiological data and outcomes in digital biomarkers and concluded that this tool has the potential to be used as a system for recognizing and categorizing behavioral and physiological data in the field. As there is no categorization scheme for digital biomarkers, we propose that researchers use this tool. This review also used other coding systems such as the ICD-11 and ICHI. The former allows systematic classification of the population, whereas the latter supports categorization of interventions. Using these systematized techniques, we can place digital biomarker research into a relevant population, intervention, comparison, and outcomes query. This may help to ensure the consistency and advancement of digital biomarker research [86] because the new Medical Device Regulation in the European Union [87] has increased the need for clinical evidence to support medical device approval; hence, the number of industry-sponsored SRs in this area is expected to increase in the future.

### Implications

This scoping review examined SRs of digital biomarker–based studies regarding population, intervention, and outcomes. To our knowledge, this is the first scoping review of SRs of RCTs involving digital biomarkers. Therefore, these results may help clinicians and researchers to keep updated about the scope of the literature concerning digital biomarkers. In addition, we highlighted the behavioral and physiological data types as well as digital devices and sensors used in SRs of digital biomarkers. The aforementioned findings could also inform researchers about the field’s gaps, as examined in the Digital Devices, Physiological and Behavioral Data, and Sensors subsection the Discussion section. In addition, as mentioned earlier, we have proposed that the ICF tool can be used by digital biomarker researchers as a standard tool for categorizing behavioral and physiological data.

### Limitations

This study’s findings should be considered in light of its limitations. First, we searched for studies on digital biomarkers using a mix of “digital biomarkers, wearable, implantable, portable, digestible” terms. We did not test the search strategy’s precision and specificity in finding all field-related research, but we hypothesized that relevant publications might be found using this method. Nonetheless, this search formula may have missed some SRs relating to this topic. Second, the short time period (2019-2020) of the study is one of its possible weaknesses. Due to the broad scope of the topic, we chose a shorter time period. However, given the new European medical device legislation proposed in 2017 [87], we felt that this would be a critical time period for reviewing clinical data before regulation.

Moreover, we expected that because the studies in our analysis were SRs, we could incorporate all relevant studies if we included SRs. It is possible that non–English-language reviews were ignored because we limited the scope of this study to English-language research. Finally, the ICHI and ICF tools used in this study to categorize interventions and outcomes are not officially authorized (they are still being developed by the WHO) because there are no established definition systems for digital technologies. We did not examine the SRs for overlap among RCTs. Therefore, some results may overlap.

### Strengths

To our knowledge, our scoping review is the most thorough presentation of SRs of digital biomarkers. Several scoping reviews have already been published on digital biomarkers, such as the use of accelerometers to measure physical activity [88], the use of wearable and mobile technology to measure and promote healthy sleep patterns in adolescents [89], the use of wearable inertial sensors in work-related activities [90], and the use of wearable sensor technology to detect shock impacts in sports and occupational settings [91], all of which relate to a specific type of digital device or population. By contrast, this scoping review was conducted as comprehensive research to demonstrate the full spectrum of the topic and how digital biomarkers are already being integrated into health care systems. Our research described the scope of SRs of digital biomarkers without limitation to a specific type of patient or digital biomarker. Another strength of our study is that we used the WHO’s classification tools (ICD-11, ICHI, and ICF) to identify and categorize the included studies’ patients, interventions, and outcomes. In addition, physiological and behavioral data (digital biomarkers) were classified using the ICF tool, which was considered reliable for this purpose.

### Conclusions

Our scoping review revealed that clinical evidence for a wide range of study populations, interventions, digital biomarkers, and sensor technologies has been systematically reviewed in recent years. Still, some clinical areas dominate, and notable unexplored fields exist. Understanding the clinical value of digital biomarkers requires a systematic assessment of the strength and quality of the evidence for their health effects. Understanding the breadth and quality of clinical evidence will inform clinical and health policy decision-makers about which areas are ripe for widespread adoption and evidence-based use of digital biomarkers and in which areas evidence gaps remain to be filled. Given the volume of literature on digital biomarkers across many health domains, specific definitions, standards, and methods for integration seem to be needed. We used the ICF tool to categorize behavioral and physiological data (digital biomarkers) in this study because there is no standard measurement in this area. The results suggest that this approach’s categorization of behavioral and physiological data is applicable to digital biomarkers.

## Appendix

Multimedia Appendix 1 Search strategy.

Multimedia Appendix 2 Systematic reviews that were excluded because of the study design.

Multimedia Appendix 3 Characteristic of the included studies.

Multimedia Appendix 4 List of excluded studies with reasons for exclusion.

Multimedia Appendix 5 Roles and types of digital biomarkers, sensors, and physiological and behavioral data, as well as digital devices.

Multimedia Appendix 6 Physiological and behavioral data categories using the International Classification of Functioning, Disability, and Health tool.

## Abbreviations

ICD-11: International Classification of Diseases, Eleventh Revision
ICF: International Classification of Functioning, Disability, and Health
ICHI: International Classification of Health Interventions
PRISMA: Preferred Reporting Items for Systematic Reviews and Meta-Analyses
PRISMA-ScR: Preferred Reporting Items for Systematic Reviews and Meta-Analyses extension for Scoping Reviews
RCT: randomized controlled trial
SR: systematic review
WHO: World Health Organization
