# Supplementary material for: Digital Biomarker–Based Studies: Scoping Review of Systematic Reviews
Source: JMIR Mhealth Uhealth. 2022 Oct 24;10(10):e35722. doi: 10.2196/35722 (PMC9641516; doi:10.2196/35722)
Supplement: Multimedia Appendix 1 [file mhealth_v10i10e35722_app1.docx]

Multimedia Appendix 1

Search formula in PubMed

| Terms | Number | Syntax |
| --- | --- | --- |
| Digital biomarkers | #1 | ("digital biomarker"[Title/Abstract] OR "digital biomarkers"[Title/Abstract] OR "portable"[Title/Abstract] OR "portables"[Title/Abstract] OR "wearable"[Title/Abstract] OR "wearables"[Title/Abstract] OR "implantable"[Title/Abstract] OR "implantables"[Title/Abstract] OR "digestible"[Title/Abstract] OR "digestibles"[Title/Abstract]) |
| Systematic reviews | #2 | (((systematic review[ti] OR systematic literature review[ti] OR systematic scoping review[ti] OR systematic narrative review[ti] OR systematic qualitative review[ti] OR systematic evidence review[ti] OR systematic quantitative review[ti] OR systematic meta-review[ti] OR systematic critical review[ti] OR systematic mixed studies review[ti] OR systematic mapping review[ti] OR systematic Cochrane review[ti] OR systematic search and review[ti] OR systematic integrative review[ti]) NOT comment[pt] NOT (protocol[ti] OR protocols[ti])) NOT MEDLINE [subset]) OR (Cochrane Database Syst Rev[ta] AND review[pt]) OR systematic review[pt] |
| Publication date | #3 | **(“2019/01/01”[Date - Publication]: “2020/12/31”[Date - Publication])** |
| Final search strategy | #4 | #1 AND #2 AND #3 |

Search formula in the Cochrane Library:

“digital biomarker” OR “digital biomarkers” OR portable OR portables OR wearable OR wearables OR implantable OR implantables OR digestible OR digestibles in Title Abstract Keyword - with Cochrane Library publication date Between Jan 2019 and Dec 2020, in Cochrane Reviews (Word variations have been searched)
