# Supplementary material for: Digital Biomarker–Based Studies: Scoping Review of Systematic Reviews
Source: JMIR Mhealth Uhealth. 2022 Oct 24;10(10):e35722. doi: 10.2196/35722 (PMC9641516; doi:10.2196/35722)
Supplement: Multimedia Appendix 2 [file mhealth_v10i10e35722_app2.docx]

| 1 | Comparison of wearable sensor to traditional methods in functional outcome measures: A systematic review [1] |
| --- | --- |
| 2 | Implantable cardioverter-defibrillators in cardiac transplant recipients: A systematic review from the Electrophysiology Collaborative Consortium for Meta-analysis-ELECTRAM investigators [2] |
| 3 | Risk stratification using late gadolinium enhancement on cardiac magnetic resonance imaging in patients with hypertrophic cardiomyopathy: A systematic review and meta-analysis [3] |
| 4 | Motion Capture Technology in Industrial Applications: A Systematic Review [4] |
| 5 | A Systematic Review of the Spectrum of Cardiac Arrhythmias in Sub-Saharan Africa [5] |
| 6 | Diagnostic accuracy of smart gadgets/wearable devices in detecting atrial fibrillation: A systematic review and meta-analysis [6] |
| 7 | Association between fine particulate matter and atrial fibrillation in implantable cardioverter defibrillator patients: a systematic review and meta-analysis [7] |
| 8 | Reliability and Validity of Commercially Available Wearable Devices for Measuring Steps, Energy Expenditure, and Heart Rate: Systematic Review [8] |
| 9 | Electromagnetic interference effect of dental equipment on cardiac implantable electrical devices: A systematic review [9] |
| 10 | The Potential of Functional Near-Infrared Spectroscopy-Based Neurofeedback-A Systematic Review and Recommendations for Best Practice [10] |
| 11 | Systematic Review of Digital Phenotyping and Machine Learning in Psychosis Spectrum Illnesses [11] |
| 12 | Wearable Transdermal Alcohol Monitors: A Systematic Review of Detection Validity, and Relationship Between Transdermal and Breath Alcohol Concentration and Influencing Factors [12] |
| 13 | Enabling Older Adults' Health Self-Management through Self-Report and Visualization-A Systematic Literature Review [13] |
| 14 | Evaluation of mHealth Applications Related to Cardiovascular Diseases: a Systematic Review [14] |
| 15 | Unmet device reprogramming needs at the end of life among patients with implantable cardioverter defibrillator: A systematic review and meta-analysis [15] |
| 16 | Validity of Wrist-Worn photoplethysmography devices to measure heart rate: A systematic review and meta-analysis [16] |
| 17 | Diagnosis of Infective Endocarditis by Subtype Using (18)F-Fluorodeoxyglucose Positron Emission Tomography/Computed Tomography: A Contemporary Meta-Analysis [17] |
| 18 | Cost-Effectiveness of Extended Electrocardiogram Monitoring for Atrial Fibrillation After Stroke: A Systematic Review [18] |
| 19 | Current Evidence for Continuous Vital Signs Monitoring by Wearable Wireless Devices in Hospitalized Adults: Systematic Review [19] |
| 20 | Objective measurement of sleep, heart rate, heart rate variability, and physical activity in suicidality: A systematic review [20] |
| 21 | Validity and reliability of wearable inertial sensors in healthy adult walking: a systematic review and meta-analysis [21] |
| 22 | Effect of ICD implantation on cardiovascular outcomes in patients with cardiac amyloidosis: A systematic review and meta-anaylsis [22] |
| 23 | Wearable Inertial Sensors to Assess Gait during the 6-Minute Walk Test: A Systematic Review [23] |
| 24 | Subcutaneous implantable cardioverter-defibrillator troubleshooting in patients with a left ventricular assist device: A case series and systematic review [24] |
| 25 | Predicting sustained ventricular arrhythmias in dilated cardiomyopathy: a meta-analysis and systematic review [25] |
| 26 | Evaluative Research of Technologies for Prehospital Communication and Coordination: a Systematic Review [26] |
| 27 | The current use of wearable sensors to enhance safety and performance in breath-hold diving: A systematic review [27] |
| 28 | Current Trends and Confounding Factors in Myoelectric Control: Limb Position and Contraction Intensity [28] |
| 29 | The prevalence of depression in patients living with implantable cardioverter defibrillator: a systematic review and meta-analysis [29] |
| 30 | Role of Wearable Accelerometer Devices in Delirium Studies: A Systematic Review [30] |
| 31 | Wide QRS complex and the risk of major arrhythmic events in Brugada syndrome patients: A systematic review and meta-analysis [31] |
| 32 | Objectively Measured Physical Activity, Sedentary Behavior, and Metabolic Syndrome in Adults: Systematic Review of Observational Evidence [32] |
| 33 | Magnetic resonance imaging in non-conditional pacemakers and implantable cardioverter-defibrillators: a systematic review and meta-analysis [33] |
| 34 | Analysis of Relevant Features from Photoplethysmographic Signals for Atrial Fibrillation Classification [34] |
| 35 | A systematic review of the accuracy of sleep wearable devices for estimating sleep onset [35] |
| 36 | Accuracy of Wristband Fitbit Models in Assessing Sleep: Systematic Review and Meta-Analysis [36] |
| 37 | Use of Near-infrared Spectroscopy and Implantable Doppler for Postoperative Monitoring of Free Tissue Transfer for Breast Reconstruction: A Systematic Review and Meta-analysis [37] |
| 38 | Are cost-effective technologies feasible to measure gait in older adults? A systematic review of evidence-based literature [38] |
| 39 | A Systematic Review of Evidence for a Role of Rest-Activity Rhythms in Dementia [39] |
| 40 | Wearable systems for shoulder kinematics assessment: a systematic review [40] |
| 41 | Prognostic Value of Late Gadolinium Enhancement in Predicting Life-Threatening Arrhythmias in Heart Failure Patients With Implantable Cardioverter-Defibrillators: A Systematic Review and Meta-Analysis [41] |
| 42 | Wearable Sensors for Monitoring of Cigarette Smoking in Free-Living: A Systematic Review [42] |
| 43 | Systematic Review and Meta-Analysis of Clinical Outcome After Implantable Cardioverter-Defibrillator Therapy in Patients With Chagas Heart Disease [43] |
| 44 | Association Between Temporal Spatial Parameters and Overuse Injury History in Runners: A Systematic Review and Meta-analysis [44] |
| 45 | Predictors of Late Mortality in D-Transposition of the Great Arteries After Atrial Switch Repair: Systematic Review and Meta-Analysis [45] |
| 46 | Wearable sensors, cerebral palsy and gait assessment in everyday environments: is it a reality? - A systematic review [46] |
| 47 | A Systematic Review on the Value of Infrared Thermography in the Early Detection of Periprosthetic Joint Infections [47] |
| 48 | Analyzing the Use of Accelerometers as a Method of Early Diagnosis of Alterations in Balance in Elderly People: A Systematic Review [48] |
| 49 | Current State of Digital Biomarker Technologies for Real-Life, Home-Based Monitoring of Cognitive Function for Mild Cognitive Impairment to Mild Alzheimer Disease and Implications for Clinical Care: Systematic Review [49] |
| 50 | Subcutaneous implantable cardioverter-defibrillator in primary and secondary prevention of sudden cardiac death: A meta-analysis [50] |
| 51 | A Systematic Review and Implementation of IoT-Based Pervasive Sensor-Enabled Tracking System for Dementia Patients [51] |
| 52 | A systematic review of feasibility studies promoting the use of mobile technologies in clinical research [52] |
| 53 | Quantification of the validity and reliability of sprint performance metrics computed using inertial sensors: A systematic review [53] |
| 54 | Pooled Analysis of the Efficacy and Safety of Video Capsule Endoscopy in Patients with Implantable Cardiac Devices [54] |
| 55 | Toe Pressure in Predicting Diabetic Foot Ulcer Healing: A Systematic Review and Meta-analysis [55] |
| 56 | A Systematic Review of Wearable Sensors and IoT-Based Monitoring Applications for Older Adults - a Focus on Ageing Population and Independent Living [56] |
| 57 | Assessing Eating Behaviour Using Upper Limb Mounted Motion Sensors: A Systematic Review [57] |
| 58 | Association between CRT(D)/ICD and renal insufficiency: A systematic review and meta-analysis [58] |
| 59 | Wearable health technology to quantify the functional impact of peripheral neuropathy on mobility in Parkinson’s disease: A systematic review [59] |
| 60 | Biofeedback systems for gait rehabilitation of individuals with lower-limb amputation: A systematic review [60] |
| 61 | The Use of Activity Trackers in Interventions for Childhood Cancer Patients and Survivors: A Systematic Review [61] |
| 62 | Predefined vs data-guided training prescription based on autonomic nervous system variation: A systematic review [62] |
| 63 | Current reporting of usability and impact of mHealth interventions for substance use disorder: A systematic review [63] |
| 64 | Effects of real-time feedback on cardiopulmonary resuscitation quality on outcomes in adult patients with cardiac arrest: A systematic review and meta-analysis [64] |
| 65 | Effects of Motivational Interviewing and Wearable Fitness Trackers on Motivation and Physical Activity: A Systematic Review [65] |
| 66 | Diagnostic Performance of Prehospital Point-of-Care Troponin Tests to Rule out Acute Myocardial Infarction: A Systematic Review [66] |
| 67 | The role of an antibiotic envelope in the prevention of major cardiac implantable electronic device infections: A systematic review and meta-analysis [67] |
| 68 | Efficacy of antibacterial envelope in prevention of cardiovascular implantable electronic device infections in high-risk patients: A systematic review and meta-analysis [68] |
| 69 | Long-term weight management using wearable technology in overweight and obese adults: Systematic review [69] |
| 70 | Sensing technology to facilitate behavioral and psychological symptoms and to monitor treatment response in people with dementia: A systematic review [70] |
| 71 | Association of Exposures to Seated Postures With Immediate Increases in Back Pain: A Systematic Review of Studies With Objectively Measured Sitting Time [71] |
| 72 | Antibiotic envelope is associated with reduction in cardiac implantable electronic devices infections especially for high-power device—Systematic review and meta-analysis [72] |
| 73 | Implantable cardiac monitors to detect atrial fibrillation after cryptogenic stroke: A systematic review and economic evaluation [73] |
| 74 | Outcomes following implantable cardioverter–defibrillator generator replacement in adults: A systematic review [74] |
| 75 | Can Mobile Technology Improve Weight Loss in Overweight Adults? A Systematic Review [75] |
| 76 | Time trends in sudden cardiac death risk in heart failure patients with cardiac resynchronization therapy: A systematic review [76] |
| 77 | Device monitoring in heart failure management: Outcomes based on a systematic review and meta-analysis [77] |
| 78 | The Role of Technology in Adherence to Physical Activity Programs in Patients with Chronic Diseases Experiencing Fatigue: a Systematic Review [78] |
| 79 | Exercise Interventions in Patients with Implantable Cardioverter-Defibrillators and Cardiac Resynchronization Therapy: A SYSTEMATIC REVIEW and META-ANALYSIS [79] |
| 80 | Patient generated health data use in clinical practice: A systematic review [80] |
| 81 | Frailty, Implantable Cardioverter Defibrillators, and Mortality: a Systematic Review [81] |
| 82 | A Systematic Review of Electronic Health (eHealth) interventions to improve physical activity in patients with breast cancer [82] |
| 83 | Mobile health interventions to promote physical activity and reduce sedentary behaviour in the workplace: A systematic review [83] |
| 84 | Device infections in implantable cardioverter defibrillators versus permanent pacemakers: A systematic review and meta-analysis [84] |
| 85 | Data management and wearables in older adults: A systematic review.[85] |
| 86 | Wearable Cardioverter-Defibrillator Therapy for the Prevention of Sudden Cardiac Death: A Systematic Review and Meta-Analysis [86] |
| 87 | Sleep Tracking: a Systematic Review of the Research Using Commercially Available Technology [87] |
| 88 | Association between atrial fibrillation and patient-important outcomes in heart failure patients with implantable cardioverter-defibrillators: A systematic review and meta-analysis [88] |
| 89 | Cardiac resynchronization therapy and outcomes in patients with left ventricular assist devices: a systematic review and meta-analysis [89] |

References

1. Follis S, Chen Z, Mishra S, Howe CL, Toosizadeh N, Dohm M. Comparison of wearable sensor to traditional methods in functional outcome measures: A systematic review. J Orthop Res 2021;39(10):2093–2102. PMID:33300119

2. Garg J, Shah K, Turagam MK, Tzou W, Gopinathannair R, Natale A, Lakkireddy D. Implantable cardioverter‐defibrillators in cardiac transplant recipients: A systematic review from the Electrophysiology Collaborative Consortium for Meta‐analysis—ELECTRAM investigators. Pacing Clin Electrophysiol [Internet] 2020 Dec 12;43(12):1529–1537. [doi: 10.1111/pace.14098]

3. Kamp NJ, Chery G, Kosinski AS, Desai MY, Wazni O, Schmidler GS, Patel M, Lopes RD, Morin DP, Al-Khatib SM. Risk stratification using late gadolinium enhancement on cardiac magnetic resonance imaging in patients with hypertrophic cardiomyopathy: A systematic review and meta-analysis. Prog Cardiovasc Dis [Internet] Elsevier Inc.; 2021;66:10–16. PMID:33171204

4. Menolotto M, Komaris DS, Tedesco S, O’flynn B, Walsh M. Motion capture technology in industrial applications: A systematic review. Sensors (Switzerland) 2020;20(19):1–25. PMID:33028042

5. Yuyun MF, Bonny A, Ng GA, Sliwa K, Kengne AP, Chin A, Mocumbi AO, Ngantcha M, Ajijola OA, Bukhman G. A systematic review of the spectrum of cardiac arrhythmias in Sub-Saharan Africa. Glob Heart 2020;15(1). PMID:32923331

6. Prasitlumkum N, Cheungpasitporn W, Chokesuwattanaskul A, Thangjui S, Thongprayoon C, Bathini T, Vallabhajosyula S, Kanitsoraphan C, Leesutipornchai T, Chokesuwattanaskul R. Diagnostic accuracy of smart gadgets/wearable devices in detecting atrial fibrillation: A systematic review and meta-analysis. Arch Cardiovasc Dis [Internet] Elsevier Masson SAS; 2021;114(1):4–16. PMID:32921618

7. Yue C, Yang F, Wang L, Li F, Chen Y. Association between fine particulate matter and atrial fibrillation in implantable cardioverter defibrillator patients: a systematic review and meta-analysis. J Interv Card Electrophysiol Journal of Interventional Cardiac Electrophysiology; 2020;59(3):595–601. PMID:32918184

8. Fuller D, Colwell E, Low J, Orychock K, Ann Tobin M, Simango B, Buote R, van Heerden D, Luan H, Cullen K, Slade L, Taylor NGA. Reliability and Validity of Commercially Available Wearable Devices for Measuring Steps, Energy Expenditure, and Heart Rate: Systematic Review. JMIR mHealth uHealth 2020;8(9):1–23. PMID:32897239

9. Niu Y, Chen Y, Li W, Xie R, Deng X. Electromagnetic interference effect of dental equipment on cardiac implantable electrical devices: A systematic review. PACE - Pacing Clin Electrophysiol 2020;43(12):1588–1598. PMID:32852847

10. Kohl SH, Mehler DMA, Lührs M, Thibault RT, Konrad K, Sorger B. The Potential of Functional Near-Infrared Spectroscopy-Based Neurofeedback—A Systematic Review and Recommendations for Best Practice. Front Neurosci 2020;14(July). [doi: 10.3389/fnins.2020.00594]

11. Benoit J, Onyeaka H, Keshavan M, Torous J. Systematic Review of Digital Phenotyping and Machine Learning in Psychosis Spectrum Illnesses. Harv Rev Psychiatry 2020;28(5):296–304. PMID:32796192

12. van Egmond K, Wright CJC, Livingston M, Kuntsche E. Wearable Transdermal Alcohol Monitors: A Systematic Review of Detection Validity, and Relationship Between Transdermal and Breath Alcohol Concentration and Influencing Factors. Alcohol Clin Exp Res 2020;44(10):1918–1932. PMID:32767791

13. Cajamarca G, Herskovic V, Rossel PO. Enabling older adults’ health self-management through self-report and visualization—a systematic literature review†. Sensors (Switzerland) 2020;20(15):1–16. PMID:32759801

14. Villarreal V, Berbey-Alvarez A. Evaluation of mhealth applications related to cardiovascular diseases: A systematic review. Acta Inform Medica 2020;28(2):130–137. [doi: 10.5455/aim.2020.28.130-137]

15. Gonzalez-Jaramillo V, Sobanski P, Calvache JA, Arenas-Ochoa LF, Franco OH, Hunziker L, Eychmüller S, Maessen M. Unmet device reprogramming needs at the end of life among patients with implantable cardioverter defibrillator: A systematic review and meta-analysis. Palliat Med 2020;34(8):1019–1029. PMID:32588755

16. Zhang Y, Weaver RG, Armstrong B, Burkart S, Zhang S, Beets MW. Validity of Wrist-Worn photoplethysmography devices to measure heart rate: A systematic review and meta-analysis. J Sports Sci [Internet] Routledge; 2020;38(17):2021–2034. PMID:32552580

17. Wang TKM, Sánchez-Nadales A, Igbinomwanhia E, Cremer P, Griffin B, Xu B. Diagnosis of Infective Endocarditis by Subtype Using 18F-Fluorodeoxyglucose Positron Emission Tomography/Computed Tomography: A Contemporary Meta-Analysis. Circ Cardiovasc Imaging 2020;(June):1–11. PMID:32507019

18. Chew DS, Rennert-May E, Spackman E, Mark DB, Exner D V. Cost-effectiveness of extended electrocardiogram monitoring for atrial fibrillation after stroke a systematic review. Stroke 2020;51(7):2244–2248. PMID:32498661

19. Leenen JPL, Leerentveld C, van Dijk JD, van Westreenen HL, Schoonhoven L, Patijn GA. Current evidence for continuous vital signs monitoring by wearable wireless devices in hospitalized adults: Systematic review. J Med Internet Res 2020;22(6). PMID:32469323

20. Butz AM, Christopher S. von Bartheld JB and SH-H. 乳鼠心肌提取 HHS Public Access. Physiol Behav 2017;176(12):139–148. [doi: 10.1016/j.jad.2020.03.096.Objective]

21. Kobsar D, Charlton JM, Tse CTF, Esculier JF, Graffos A, Krowchuk NM, Thatcher D, Hunt MA. Validity and reliability of wearable inertial sensors in healthy adult walking: A systematic review and meta-analysis. J Neuroeng Rehabil Journal of NeuroEngineering and Rehabilitation; 2020;17(1):1–21. PMID:32393301

22. Halawa A, Woldu HG, Kacey KG, Alpert MA. Effect of ICD implantation on cardiovascular outcomes in patients with cardiac amyloidosis: A systematic review and meta-anaylsis. J Cardiovasc Electrophysiol 2020;31(7):1749–1758. PMID:32391952

23. Storm FA, Cesareo A, Reni G, Biffi E. Wearable inertial sensors to assess gait during the 6-minute walk test: A systematic review. Sensors (Switzerland) 2020;20(9). PMID:32384806

24. Black-Maier E, Lewis RK, Barnett AS, Pokorney SD, Sun AY, Koontz JI, Daubert JP, Piccini JP. Subcutaneous implantable cardioverter-defibrillator troubleshooting in patients with a left ventricular assist device: A case series and systematic review. Hear Rhythm [Internet] Heart Rhythm Society; 2020;17(9):1536–1544. PMID:32304733

25. Sammani A, Kayvanpour E, Bosman LP, Sedaghat-Hamedani F, Proctor T, Gi WT, Broezel A, Jensen K, Katus HA, te Riele ASJM, Meder B, Asselbergs FW. Predicting sustained ventricular arrhythmias in dilated cardiomyopathy: a meta-analysis and systematic review. ESC Hear Fail 2020;7(4):1430–1441. PMID:32285648

26. Zhang Z, Brazil J, Ozkaynak M, Desanto K. Evaluative Research of Technologies for Prehospital Communication and Coordination: a Systematic Review. J Med Syst 2020;44(5). PMID:32246206

27. Vinetti G, Lopomo NF, Taboni A, Fagoni N, Ferretti G. The current use of wearable sensors to enhance safety and performance in breath-hold diving: A systematic review. Diving Hyperb Med 2020;50(1):54–65. PMID:32187619

28. Campbell E, Phinyomark A, Scheme E. Current trends and confounding factors in myoelectric control: Limb position and contraction intensity. Sensors (Switzerland) 2020;20(6):1–44. PMID:32183215

29. Oshvandi K, Khatiban M, Ghanei Gheshlagh R, Razavi M. The prevalence of depression in patients living with implantable cardioverter defibrillator: a systematic review and meta-analysis. Ir J Med Sci Irish Journal of Medical Science (1971 -); 2020;189(4):1243–1252. PMID:32172313

30. Davoudi A, Manini TM, Bihorac A, Rashidi P. Role of Wearable Accelerometer Devices in Delirium Studies. Crit Care Explor 2019;1(9):e0027. [doi: 10.1097/cce.0000000000000027]

31. Rattanawong P, Kewcharoen J, Techorueangwiwat C, Kanitsoraphan C, Mekritthikrai R, Prasitlumkum N, Puttapiban P, Mekraksakit P, Vutthikraivit W, Sorajja D. Wide QRS complex and the risk of major arrhythmic events in Brugada syndrome patients: A systematic review and meta-analysis. J Arrhythmia 2020;36(1):143–152. [doi: 10.1002/joa3.12290]

32. Amirfaiz S, Shahril MR. Objectively Measured Physical Activity, Sedentary Behavior, and Metabolic Syndrome in Adults: Systematic Review of Observational Evidence. Metab Syndr Relat Disord 2019;17(1):1–21. PMID:30272527

33. Munawar DA, Chan JEZ, Emami M, Kadhim K, Khokhar K, O’shea C, Iwai S, Pitman B, Linz D, Munawar M, Roberts-Thomson K, Young GD, Mahajan R, Sanders P, Lau DH. Magnetic resonance imaging in non-conditional pacemakers and implantable cardioverter-defibrillators: A systematic review and meta-analysis. Europace 2020;22(2):288–298. PMID:31995177

34. Millán CA, Girón NA, Lopez DM. Analysis of relevant features from photoplethysmographic signals for atrial fibrillation classification. Int J Environ Res Public Health 2020;17(2). PMID:31941071

35. Scott H, Lack L, Lovato N. A systematic review of the accuracy of sleep wearable devices for estimating sleep onset. Sleep Med Rev [Internet] Elsevier Ltd; 2020;49:101227. PMID:31901524

36. Haghayegh S, Khoshnevis S, Smolensky MH, Diller KR, Castriotta RJ. Accuracy of wristband fitbit models in assessing sleep: Systematic review and meta-analysis. J Med Internet Res 2019;21(11). PMID:31778122

37. Berthelot M, Ashcroft J, Boshier P, Hunter J, Henry FP, Lo B, Yang GZ, Leff D. Use of Near-infrared Spectroscopy and Implantable Doppler for Postoperative Monitoring of Free Tissue Transfer for Breast Reconstruction: A Systematic Review and Meta-analysis. Plast Reconstr Surg - Glob Open 2019;7(10):E2437. [doi: 10.1097/GOX.0000000000002437]

38. Zhong R, Rau PLP. Are cost-effective technologies feasible to measure gait in older adults? A systematic review of evidence-based literature. Arch Gerontol Geriatr [Internet] Elsevier; 2020;87(October 2019):103970. PMID:31743825

39. Smagula SF, Gujral S, Capps CS, Krafty RT. A systematic review of evidence for a role of rest-activity rhythms in dementia. Front Psychiatry 2019;10(OCT):1–7. [doi: 10.3389/fpsyt.2019.00778]

40. Carnevale A, Longo UG, Schena E, Massaroni C, Lo Presti D, Berton A, Candela V, Denaro V. 肩关节运动评估的可穿戴系统:系统综述. BMC Musculoskelet Disord BMC Musculoskeletal Disorders; 2019;20(1).

41. Yue T, Chen B, Wu L, Xu J, Pu J. Prognostic Value of Late Gadolinium Enhancement in Predicting Life‐Threatening Arrhythmias in Heart Failure Patients With Implantable Cardioverter‐Defibrillators: A Systematic Review and Meta‐Analysis. J Magn Reson Imaging [Internet] 2020 May 11;51(5):1422–1439. [doi: 10.1002/jmri.26982]

42. Imtiaz MH, Ramos-Garcia RI, Wattal S, Tiffany S, Sazonov E. Wearable sensors for monitoring of cigarette smoking in free-living: A systematic review. Sensors (Switzerland) 2019;19(21). PMID:31661856

43. Rassi FM, Minohara L, Rassi A, Correia LCL, Marin-Neto JA, da Silva Menezes A. Systematic Review and Meta-Analysis of Clinical Outcome After Implantable Cardioverter-Defibrillator Therapy in Patients With Chagas Heart Disease. JACC Clin Electrophysiol 2019;5(10):1213–1223. PMID:31648747

44. Brindle RA, Taylor JB, Rajek C, Weisbrod A, Ford KR. Association Between Temporal Spatial Parameters and Overuse Injury History in Runners: A Systematic Review and Meta-analysis. Sport Med [Internet] Springer International Publishing; 2020;50(2):331–342. PMID:31643019

45. Venkatesh P, Evans AT, Maw AM, Pashun RA, Patel A, Kim L, Feldman D, Minutello R, Wong SC, Stribling JC, LaPar D, Holzer R, Ginns J, Bacha E, Singh HS. Predictors of Late Mortality in D-Transposition of the Great Arteries After Atrial Switch Repair: Systematic Review and Meta-Analysis. J Am Heart Assoc 2019;8(21). PMID:31642369

46. Rozin Kleiner AF, Belgamo A, Pagnussat AS, Costa e Silva A de A, Sforza C, Rocha NACF. Wearable sensors, cerebral palsy and gait assessment in everyday environments: Is it a reality? - A systematic review. Funct Neurol 2019;34(2):85–91. PMID:31556388

47. Scheidt S, Rüwald J, Schildberg FA, Mahlein AK, Seuser A, Wirtz DC, Jacobs C. A Systematic Review on the Value of Infrared Thermography in the Early Detection of Periprosthetic Joint Infections. Z Orthop Unfall 2020;158(4):397–405. PMID:31525794

48. Leirós-Rodríguez R, García-Soidán JL, Romo-Pérez V. Analyzing the use of accelerometers as a method of early diagnosis of alterations in balance in elderly people: A systematic review. Sensors (Switzerland) 2019;19(18). PMID:31505828

49. Piau A, Wild K, Mattek N, Kaye J. Current state of digital biomarker technologies for real-life, home-based monitoring of cognitive function for mild cognitive impairment to mild Alzheimer disease and implications for clinical care: Systematic review. J Med Internet Res 2019;21(8). PMID:31471958

50. León Salas B, Trujillo-Martín MM, García García J, Ramallo Fariña Y, García Quintana A, Quirós López R, Serrano-Aguilar P. Subcutaneous implantable cardioverter-defibrillator in primary and secondary prevention of sudden cardiac death: A meta-analysis. PACE - Pacing Clin Electrophysiol 2019;42(9):1253–1268. PMID:31396970

51. Ray PP, Dash D, De D. A Systematic Review and Implementation of IoT-Based Pervasive Sensor-Enabled Tracking System for Dementia Patients. J Med Syst Journal of Medical Systems; 2019;43(9). PMID:31317281

52. Bakker JP, Goldsack JC, Clarke M, Coravos A, Geoghegan C, Godfrey A, Heasley MG, Karlin DR, Manta C, Peterson B, Ramirez E, Sheth N, Bruno A, Bullis E, Wareham K, Zimmerman N, Forrest A, Wood WA. A systematic review of feasibility studies promoting the use of mobile technologies in clinical research. npj Digit Med [Internet] Springer US; 2019;2(1). [doi: 10.1038/s41746-019-0125-x]

53. Macadam P, Cronin J, Neville J, Diewald S. Quantification of the validity and reliability of sprint performance metrics computed using inertial sensors: A systematic review. Gait Posture [Internet] Elsevier; 2019;73(July):26–38. PMID:31299501

54. Tabet R, Nassani N, Karam B, Shammaa Y, Akhrass P, Deeb L. Pooled Analysis of the Efficacy and Safety of Video Capsule Endoscopy in Patients with Implantable Cardiac Devices. Can J Gastroenterol Hepatol Hindawi; 2019;2019:2–7. PMID:31236386

55. Tay WL, Lo ZJ, Hong Q, Yong E, Chandrasekar S, Tan GWL. Toe Pressure in Predicting Diabetic Foot Ulcer Healing: A Systematic Review and Meta-analysis. Ann Vasc Surg [Internet] Elsevier Inc.; 2019;60:371–378. PMID:31220591

56. Baig MM, Afifi S, GholamHosseini H, Mirza F. A Systematic Review of Wearable Sensors and IoT-Based Monitoring Applications for Older Adults – a Focus on Ageing Population and Independent Living. J Med Syst 2019;43(8):10916. PMID:31203472

57. Heydarian H, Adam M, Burrows T, Collins C, Rollo ME. Assessing eating behaviour using upper limb mounted motion sensors: A systematic review. Nutrients 2019;11(5). PMID:31137677

58. Liu Y, Sun JY, Zhu YS, Li ZM, Li KL, Wang RX. Association between CRT(D)/ICD and renal insufficiency: A systematic review and meta-analysis. Semin Dial 2021;34(1):17–30. PMID:33296540

59. Corrà MF, Warmerdam E, Vila-Chã N, Maetzler W, Maia L. Wearable health technology to quantify the functional impact of peripheral neuropathy on mobility in Parkinson’s disease: A systematic review. Sensors (Switzerland) 2020;20(22):1–34. PMID:33228056

60. Escamilla-Nunez R, Michelini A, Andrysek J. Biofeedback systems for gait rehabilitation of individuals with lower-limb amputation: A systematic review. Sensors (Switzerland) 2020;20(6). PMID:32183338

61. Ha L, Mizrahi D, Wakefield CE, Cohn RJ, Simar D, Signorelli C. The Use of Activity Trackers in Interventions for Childhood Cancer Patients and Survivors: A Systematic Review. J Adolesc Young Adult Oncol 2021;10(1):1–14. PMID:32897805

62. Düking P, Zinner C, Reed JL, Holmberg HC, Sperlich B. Predefined vs data-guided training prescription based on autonomic nervous system variation: A systematic review. Scand J Med Sci Sport 2020;30(12):2291–2304. PMID:32785959

63. Carreiro S, Newcomb M, Leach R, Ostrowski S, Boudreaux ED, Amante D. Current reporting of usability and impact of mHealth interventions for substance use disorder: A systematic review. Drug Alcohol Depend [Internet] Elsevier; 2020;215(August):108201. PMID:32777691

64. Wang SA, Su CP, Fan HY, Hou WH, Chen YC. Effects of real-time feedback on cardiopulmonary resuscitation quality on outcomes in adult patients with cardiac arrest: A systematic review and meta-analysis. Resuscitation [Internet] European Resuscitation Council, American Heart Association, Inc., and International Liaison Committee on Resuscitation.~Published by Elsevier Ireland Ltd; 2020;155(252):82–90. PMID:32755666

65. Nuss K, Moore K, Nelson T, Li K. Effects of Motivational Interviewing and Wearable Fitness Trackers on Motivation and Physical Activity: A Systematic Review. Am J Heal Promot 2020;1–10. [doi: 10.1177/0890117120939030]

66. Alghamdi A, Alotaibi A, Alharbi M, Reynard C, Body R. Diagnostic Performance of Prehospital Point-of-Care Troponin Tests to Rule out Acute Myocardial Infarction: A Systematic Review. Prehosp Disaster Med 2020;35(5):567–573. PMID:32641173

67. Asbeutah AAA, Salem MH, Asbeutah SA, Abu-Assi MA. The role of an antibiotic envelope in the prevention of major cardiac implantable electronic device infections: A systematic review and meta-analysis. Medicine (Baltimore) 2020;99(26):e20834. PMID:32590773

68. Ullah W, Nadeem N, Haq S, Thelmo FL, Abdullah HM, Haas DC. Efficacy of antibacterial envelope in prevention of cardiovascular implantable electronic device infections in high-risk patients: A systematic review and meta-analysis. Int J Cardiol [Internet] Elsevier B.V.; 2020;315(xxxx):51–56. PMID:32291170

69. Fawcett E, van Velthoven MH, Meinert E. Long-term weight management using wearable technology in overweight and obese adults: Systematic review. JMIR mHealth uHealth 2020;8(3):1–10. PMID:32154788

70. Husebo BS, Heintz HL, Berge LI, Owoyemi P, Rahman AT, Vahia I V. Sensing technology to facilitate behavioral and psychological symptoms and to monitor treatment response in people with dementia: A systematic review. Front Pharmacol 2020;10(February):1–13. [doi: 10.3389/fphar.2019.01699]

71. De Carvalho DE, de Luca K, Funabashi M, Breen A, Wong AYL, Johansson MS, Ferreira ML, Swab M, Neil Kawchuk G, Adams J, Hartvigsen J. Association of Exposures to Seated Postures With Immediate Increases in Back Pain: A Systematic Review of Studies With Objectively Measured Sitting Time. J Manipulative Physiol Ther [Internet] Elsevier Ltd; 2020;43(1):1–12. PMID:32081511

72. Pranata R, Tondas AE, Vania R, Yuniadi Y. Antibiotic envelope is associated with reduction in cardiac implantable electronic devices infections especially for high-power device—Systematic review and meta-analysis. J Arrhythmia 2020;36(1):166–173. [doi: 10.1002/joa3.12270]

73. Edwards SJ, Wakefield V, Jhita T, Kew K, Cain P, Marceniuk G. Implantable cardiac monitors to detect atrial fibrillation after cryptogenic stroke: A systematic review and economic evaluation. Health Technol Assess (Rockv) [Internet] 2020;24(5):v–184. PMID:31944175

74. McCarthy KJ, Locke AH, Coletti M, Young D, Merchant FM, Kramer DB. Outcomes following implantable cardioverter–defibrillator generator replacement in adults: A systematic review. Hear Rhythm [Internet] Elsevier Inc.; 2020;17(6):1036–1042. PMID:31931173

75. Wang E, Abrahamson K, Liu PJ, Ahmed A. Can Mobile Technology Improve Weight Loss in Overweight Adults? A Systematic Review. West J Nurs Res 2020;42(9):747–759. PMID:31762402

76. Barra S, Providência R, Narayanan K, Boveda S, Duehmke R, Garcia R, Leyva F, Roger V, Jouven X, Agarwal S, Levy WC, Marijon E. Time trends in sudden cardiac death risk in heart failure patients with cardiac resynchronization therapy: A systematic review. Eur Heart J 2020;41(21):1976–1986. PMID:31750896

77. Halawa A, Enezate T, Flaker G. Device monitoring in heart failure management: Outcomes based on a systematic review and meta-analysis. Cardiovasc Diagn Ther 2019;9(4):386–393. [doi: 10.21037/cdt.2019.01.02]

78. Albergoni A, Hettinga FJ, La Torre A, Bonato M, Sartor F. The Role of Technology in Adherence to Physical Activity Programs in Patients with Chronic Diseases Experiencing Fatigue: a Systematic Review. Sport Med - Open Sports Medicine - Open; 2019;5(1). [doi: 10.1186/s40798-019-0214-z]

79. Steinhaus DA, Lubitz SA, Noseworthy PA, Kramer DB. Exercise Interventions in Patients with Implantable Cardioverter-Defibrillators and Cardiac Resynchronization Therapy: A SYSTEMATIC REVIEW and META-ANALYSIS. J Cardiopulm Rehabil Prev 2019;39(5):308–317. PMID:31397767

80. Demiris G, Iribarren SJ, Sward K, Lee S, Yang R. Patient generated health data use in clinical practice: A systematic review. Nurs Outlook [Internet] Elsevier Inc.; 2019;67(4):311–330. PMID:31277895

81. Chen MY, Orkaby AR, Rosenberg MA, Driver JA. Frailty, Implantable Cardioverter Defibrillators, and Mortality: a Systematic Review. J Gen Intern Med Journal of General Internal Medicine; 2019;34(10):2224–2231. PMID:31264082

82. Dorri S, Asadi F, Olfatbakhsh A, Kazemi A. A Systematic Review of Electronic Health (eHealth) interventions to improve physical activity in patients with breast cancer. Breast Cancer [Internet] Springer Japan; 2020;27(1):25–46. PMID:31187411

83. Buckingham SA, Williams AJ, Morrissey K, Price L, Harrison J. Mobile health interventions to promote physical activity and reduce sedentary behaviour in the workplace: A systematic review. Digit Heal 2019;5:1–50. [doi: 10.1177/2055207619839883]

84. Rattanawong P, Kewcharoen J, Mekraksakit P, Mekritthikrai R, Prasitlumkum N, Vutthikraivit W, Putthapiban P, Dworkin J. Device infections in implantable cardioverter defibrillators versus permanent pacemakers: A systematic review and meta-analysis. J Cardiovasc Electrophysiol 2019;30(7):1053–1065. PMID:30938929

85. Alharbi M, Straiton N, Smith S, Neubeck L, Gallagher R. Data management and wearables in older adults: A systematic review. Maturitas [Internet] Elsevier; 2019;124(February):100–110. PMID:30910279

86. Masri A, Altibi AM, Erqou S, Zmaili MA, Saleh A, Al-Adham R, Ayoub K, Baghal M, Alkukhun L, Barakat AF, Jain S, Saba S, Adelstein E. Wearable Cardioverter-Defibrillator Therapy for the Prevention of Sudden Cardiac Death: A Systematic Review and Meta-Analysis. JACC Clin Electrophysiol 2019;5(2):152–161. PMID:30784684

87. Robbins R, Seixas A, Walton Masters L, Chanko N, Diaby F, Vieira D, Jean-Louis G. Sleep Tracking: a Systematic Review of the Research Using Commercially Available Technology. Curr Sleep Med Reports Current Sleep Medicine Reports; 2019;5(3):156–163. [doi: 10.1007/s40675-019-00150-1]

88. Naka KK, Bazoukis G, Bechlioulis A, Korantzopoulos P, Michalis LK, Ntzani EE. Association between atrial fibrillation and patient-important outcomes in heart failure patients with implantable cardioverter-defibrillators: A systematic review and meta-analysis. Eur Hear J - Qual Care Clin Outcomes 2019;5(2):96–104. PMID:30462233

89. Voruganti DC, Briasoulis A, Chaudhry M, Alvarez P, Cotarlan V, Bhama JK, Giudici M. Cardiac resynchronization therapy and outcomes in patients with left ventricular assist devices: a systematic review and meta-analysis. Heart Fail Rev Heart Failure Reviews; 2019;24(2):229–236. PMID:30259285
