# Supplementary material for: Digital Biomarker–Based Studies: Scoping Review of Systematic Reviews
Source: JMIR Mhealth Uhealth. 2022 Oct 24;10(10):e35722. doi: 10.2196/35722 (PMC9641516; doi:10.2196/35722)
Supplement: Multimedia Appendix 3 [file mhealth_v10i10e35722_app3.docx]

| Author(year)/ Country | Number of included studies | Age (sex) / participants | Intervention | Comparator | Outcome(s) |
| --- | --- | --- | --- | --- | --- |
| Lu, L (2020)/ China | 82 | NS^a^ (both)/Alzheimer, Respiratory Diseases, Cardiac Conduction, Cognitive dysfunction, blindness, obstructive sleep apnea | Wearable Health Devices | NS | safety monitoring, chronic disease management, disease treatment, rehabilitation |
| Possamai, C.G (2020)/France | 75 | Adults (both)/ Diabetes, Cardiac and vascular diseases, Chronic obstructive pulmonary, Cancer, Insomnia, Renal disease, HIV/AIDS, Obstructive sleep apnea, Osteoarthritis/osteoporosis, Psychiatric disorders, Multiple sclerosis, Psoriasis, Rheumatoid arthritis, Obesity, Spinal cord injury | pharmacological or non-pharmacological | NS | glycemic control, diabetic foot complications, physical activity, blood pressure control, sleep quality, adherence to treatment, pulmonary capacity, heart rate variability |
| Ringeval, M (2020)/ Canada | 41 | Adults, older adults (both)/ cardiovascular risks, chronic obstructive pulmonary disease, cardiometabolic diseases, employees or students, such as medical students, healthy subjects | Fitbit | Without a device | Steps per day, physical activity (MVPA ^e^, min/day), weight (kg) |
| Jang, JP (2020)/ Taiwan | 32 | Older adults (both)/patients with cardiovascular implantable electronic devices such as permanent pacemakers, implantable cardioverter defibrillators, cardiac resynchronization therapy | Remote monitoring | In office follow up | Detection rate of atrial arrhythmia, incidence of stroke |
| Kamei, T (2020)/ Japan | 11 | Adults (both)/people with chronic obstructive pulmonary disease, diabetes mellitus, cardiac disease | Wearable devices | Usual care | Adherence and improve telehealth outcomes |
| Liu, JY (2020)/ Hong Kong | 10 | Older adults (both)/sedentary population | Wearable activity tracker | Without a device | Step counts, time spent on moderate-to-vigorous (MVPA) exercise |
| Tang, M.S.S (2020)/ Australia | 12 | Adults (both)/Population without clinical conditions | Wearable activity trackers | control group | Steps per day, Weight loss |
| Franssen, W.M.A (2020)/ Belgium | 35 | Adults (both)/Chronic diseases | Wearable activity trackers | Usual activity | Steps per day |
| Kwan, R.Y.C (2020)/ Hong Kong | 38 | Adults (both)/ healthy adults, Obstructive Sleep Apnea, Chronic Obstructive Disease, Cardiac disease, Obese, diabetes, Overweight, Parkinson’s disease | Wearable activity trackers | Usual care | Physical activity time, step per day |
| Burge, A.T (2020)/ Australia | 76 | Adults (both)/Chronic obstructive pulmonary disease | Wearable activity trackers | Usual care | Physical activity |
| Gama, F (2020)/ Portugal | 11 | Adults (both)/Heart failure with reduced ejection | ICD | Without ICD | All-cause mortality, udden cardiac death, all-cause death |
| Alotaibi, S (2020)/ the United Kingdom of Great Britain and Northern Ireland | 13 | Adults, elderly (both)/Heart failure | Implantable cardiac devices | Standard of care | Hospitalization, all-cause mortality |
| Lynch, C (2020)/ Australia | 21 | Adults (both)/ sedentary, office workers, Overweight, Employees, breast cancer, College students, Type 2 diabetics, osteoarthritis, peripheral artery disease, Cognitively impaired | Wearable activity trackers | Normal daily activities | Sedentary time, step count, energy expenditure |
| Kheiri, B (2019)/ United States of America | 22 | Adults, elderly (both)/Patients with ICD | Catheter Ablation | Antiarrhythmic Drugs | Tachyarrhythmias, ICD shocks, deaths |
| Hodkinson, A (2019)/ the United Kingdom of Great Britain and Northern Ireland | 36 | Adults (both)/Cardio metabolic Conditions | Monitoring devices such as pedometers and accelerometers | Usual care, | Physical activity, weight, blood glucose level, blood pressure, cholesterol levels |
| Disertori, M (2020)/ Italy | 10 | Adults, elderly (both)/Heart failure | ICD | Standard medical therapy | sudden cardiac death, primary prevention |
| Hannan, A.L (2019)/ Australia | 9 | Adults, Older adults (both)/ myocardial infarction, acute coronary syndrome, percutaneous coronary intervention, coronary artery disease, cardiac surgery (coronary artery bypass graft, valvular repair or replacement | Wearable physical activity monitoring devices with exercise prescription | Control group | Aerobic capacity, Step count, physical activity |
| Yen, H.Y (2019)/ Taiwan | 19 | Adults (both)/ Nonclinical Populations | Wearable technology | Without wearables | Weight control |
| Jo, A (2019)/ United States of America | 6 | Adults (both)/ Snoring or sleep apnea, employees, Overweight, obese, Medical residents, type 2 diabetes | Wearable watch devices | Without a device | Blood glucose level, blood pressure, cholesterol level, and weight loss |
| Tseng, A.S (2019)/ United States of America | 68 | Adults (both)/Left ventricular ejection fraction | Medical therapy alone or in combination, as well as all cardiac implantable electronic device therapies | Placebo | All-cause mortality |
| Mitchel, M.S (2020)/ Canada | 23 | Adults (both)/ Nonclinical Populations | Financial incentives | Control group | Physical activity, weight |
| Martinez, B.K (2020)/ the United Kingdom of Great Britain and Northern Ireland | 5 | Adults (both)/Ischemic heart disease implanted with an ICD | Catheter ablation | Without catheter ablation | Appropriate ICD therapies, appropriate ICD shocks, ventricular tachycardia storm, recurrent ventricular tachycardia/ventricular fibrillation (VF), cardiac hospitalizations, all-cause mortality |
| Brickwood, K.J (2019)/ Australia | 28 | Adults (both)/ Nonclinical Populations | Wearable Activity Trackers | Without a device | Steps, physical activity |
| Sypes, E.E (2019)/ Canada | 22 | NS (both)/Nonclinical Populations | Electronic Activity Monitor Systems | control group | physical activity, weight loss |
| Braakhuis, H.E.M (2019)/ Netherlands | 14 | Adults, Older adults (both)/Stroke, Coronary  artery disease  patients, Non-compliant  patients after  a cardiac  rehabilitation  programme, Chronic obstructive pulmonary disease, Inactive patients  with cardiac  diseases, Communitydwelling  elderly, Parkinson, Patients  with heart  failure and  implantable  cardioverter  defibrillator, Diabetes | Wearable activity monitors | Usual care | Physical activity, steps, walking |
| Nielsen, K.M (2019)/ Denmark | 8 | Adults (both)/Patients with cardiac implantable electronic device | Exercise-based cardiac rehabilitation | Group of no intervention, treatment as usual or another rehabilitation program with no physical exercise element) | All-cause mortality, serious adverse events, health-related quality of life, exercise capacity, antitachycardia pacing, shock, nonserious adverse events, employment or loss of employment, cost-effctiveness |
| Chan, K.L (2019)/ Hong Kong | 16 | Adults (females)/Pregnant and postpartum women | Monitoring devices | Usual care | Healthy pregnancy and maternal  well-being |
| Kirk, M.A (2019)/ Canada | 35 | Adults (both)/Cardiometabolic chronic disease | Wearable device | Exercise | Physical activity |
| Freak-Poli, R.LA (2020)/ Australia | 14 | Adults (both)/ Employed individuals | Pedometer | Without pedometer | Physical activity |
| Schaffer, K (2019)/ United States of America | 12 | Adults (both)/Cancer | Digital Activity Trackers | Usual care | Physical activity, step count |
| Armstrong, M (2019)/ Belgium | 17 | Older adults (both)/ Chronic obstructive pulmonary disease | Pedometer | Without physical activity promotion intervention | Steps per day |

^a^NS: Not speciafied, ^b^ICD: implantable cardioverter defibrillator. ^c^Non-RCT: non randomized controlled trial. ^d^CRT: Cardiac resynchronization therapy. ^e^ MVPA: Moderate-to-vigorous physical activity.
