# Supplementary material for: Digital Biomarker–Based Studies: Scoping Review of Systematic Reviews
Source: JMIR Mhealth Uhealth. 2022 Oct 24;10(10):e35722. doi: 10.2196/35722 (PMC9641516; doi:10.2196/35722)
Supplement: Multimedia Appendix 4 [file mhealth_v10i10e35722_app4.docx]

| No. | Article title | Exclusion reasons |
| --- | --- | --- |
| 1 | Systematic review of context-aware digital behavior change interventions to improve health [1] | Timeframe |
| 2 | Baseline fragmented QRS is associated with increased all-cause mortality in heart failure with reduced ejection fraction: A systematic review and meta-analysis [2] | Timeframe |
| 3 | A systematic review of effectiveness and economic evaluation of Cardiohelp and portable devices for extracorporeal membrane oxygenation (ECMO) [3] | Timeframe |
| 4 | Cephalic vs. subclavian lead implantation in cardiac implantable electronic devices: a systematic review and meta-analysis [4] | Timeframe |
| 5 | Implantable cardiac defibrillators for people with non-ischaemic cardiomyopathy [5] | Timeframe |
| 6 | Validated screening tools for the assessment of cachexia, sarcopenia, and malnutrition: a systematic review [6] | Timeframe |
| 7 | Wearable sensing devices for upper limbs: A systematic review [7] | Timeframe |
| 8 | Comparison of wearable sensor to traditional methods in functional outcome measures: A systematic review [8] | All included studies are not RCT |
| 9 | Implantable cardioverter-defibrillators in cardiac transplant recipients: A systematic review from the Electrophysiology Collaborative Consortium for Meta-analysis-ELECTRAM investigators [9] | All included studies are not RCT |
| 10 | Risk stratification using late gadolinium enhancement on cardiac magnetic resonance imaging in patients with hypertrophic cardiomyopathy: A systematic review and meta-analysis [10] | All included studies are not RCT |
| 11 | Motion Capture Technology in Industrial Applications: A Systematic Review [11] | All included studies are not RCT |
| 12 | A Systematic Review of the Spectrum of Cardiac Arrhythmias in Sub-Saharan Africa [12] | All included studies are not RCT |
| 13 | Diagnostic accuracy of smart gadgets/wearable devices in detecting atrial fibrillation: A systematic review and meta-analysis [13] | All included studies are not RCT |
| 14 | Association between fine particulate matter and atrial fibrillation in implantable cardioverter defibrillator patients: a systematic review and meta-analysis [14] | All included studies are not RCT |
| 15 | Reliability and Validity of Commercially Available Wearable Devices for Measuring Steps, Energy Expenditure, and Heart Rate: Systematic Review [15] | All included studies are not RCT |
| 16 | Electromagnetic interference effect of dental equipment on cardiac implantable electrical devices: A systematic review [16] | All included studies are not RCT |
| 17 | The Potential of Functional Near-Infrared Spectroscopy-Based Neurofeedback-A Systematic Review and Recommendations for Best Practice [17] | All included studies are not RCT |
| 18 | Systematic Review of Digital Phenotyping and Machine Learning in Psychosis Spectrum Illnesses [18] | All included studies are not RCT |
| 19 | Wearable Transdermal Alcohol Monitors: A Systematic Review of Detection Validity, and Relationship Between Transdermal and Breath Alcohol Concentration and Influencing Factors [19] | All included studies are not RCT |
| 20 | Enabling Older Adults' Health Self-Management through Self-Report and Visualization-A Systematic Literature Review [20] | All included studies are not RCT |
| 21 | Evaluation of mHealth Applications Related to Cardiovascular Diseases: a Systematic Review [21] | All included studies are not RCT |
| 22 | Unmet device reprogramming needs at the end of life among patients with implantable cardioverter defibrillator: A systematic review and meta-analysis [22] | All included studies are not RCT |
| 23 | Validity of Wrist-Worn photoplethysmography devices to measure heart rate: A systematic review and meta-analysis [23] | All included studies are not RCT |
| 24 | Diagnosis of Infective Endocarditis by Subtype Using (18)F-Fluorodeoxyglucose Positron Emission Tomography/Computed Tomography: A Contemporary Meta-Analysis [24] | All included studies are not RCT |
| 25 | Cost-Effectiveness of Extended Electrocardiogram Monitoring for Atrial Fibrillation After Stroke: A Systematic Review [25] | All included studies are not RCT |
| 26 | Current Evidence for Continuous Vital Signs Monitoring by Wearable Wireless Devices in Hospitalized Adults: Systematic Review [26] | All included studies are not RCT |
| 27 | Objective measurement of sleep, heart rate, heart rate variability, and physical activity in suicidality: A systematic review [27] | All included studies are not RCT |
| 28 | Validity and reliability of wearable inertial sensors in healthy adult walking: a systematic review and meta-analysis [28] | All included studies are not RCT |
| 29 | Effect of ICD implantation on cardiovascular outcomes in patients with cardiac amyloidosis: A systematic review and meta-anaylsis [29] | All included studies are not RCT |
| 30 | Wearable Inertial Sensors to Assess Gait during the 6-Minute Walk Test: A Systematic Review [30] | All included studies are not RCT |
| 31 | Subcutaneous implantable cardioverter-defibrillator troubleshooting in patients with a left ventricular assist device: A case series and systematic review [31] | All included studies are not RCT |
| 32 | Predicting sustained ventricular arrhythmias in dilated cardiomyopathy: a meta-analysis and systematic review [32] | All included studies are not RCT |
| 33 | Evaluative Research of Technologies for Prehospital Communication and Coordination: a Systematic Review [33] | All included studies are not RCT |
| 34 | The current use of wearable sensors to enhance safety and performance in breath-hold diving: A systematic review [34] | All included studies are not RCT |
| 35 | Current Trends and Confounding Factors in Myoelectric Control: Limb Position and Contraction Intensity [35] | All included studies are not RCT |
| 36 | The prevalence of depression in patients living with implantable cardioverter defibrillator: a systematic review and meta-analysis [36] | All included studies are not RCT |
| 37 | Role of Wearable Accelerometer Devices in Delirium Studies: A Systematic Review [37] | All included studies are not RCT |
| 38 | Wide QRS complex and the risk of major arrhythmic events in Brugada syndrome patients: A systematic review and meta-analysis [38] | All included studies are not RCT |
| 39 | Objectively Measured Physical Activity, Sedentary Behavior, and Metabolic Syndrome in Adults: Systematic Review of Observational Evidence [39] | All included studies are not RCT |
| 40 | Magnetic resonance imaging in non-conditional pacemakers and implantable cardioverter-defibrillators: a systematic review and meta-analysis [40] | All included studies are not RCT |
| 41 | Analysis of Relevant Features from Photoplethysmographic Signals for Atrial Fibrillation Classification [41] | All included studies are not RCT |
| 42 | A systematic review of the accuracy of sleep wearable devices for estimating sleep onset [42] | All included studies are not RCT |
| 43 | Accuracy of Wristband Fitbit Models in Assessing Sleep: Systematic Review and Meta-Analysis [43] | All included studies are not RCT |
| 44 | Use of Near-infrared Spectroscopy and Implantable Doppler for Postoperative Monitoring of Free Tissue Transfer for Breast Reconstruction: A Systematic Review and Meta-analysis [44] | All included studies are not RCT |
| 45 | Are cost-effective technologies feasible to measure gait in older adults? A systematic review of evidence-based literature [45] | All included studies are not RCT |
| 46 | A Systematic Review of Evidence for a Role of Rest-Activity Rhythms in Dementia [46] | All included studies are not RCT |
| 47 | Wearable systems for shoulder kinematics assessment: a systematic review [47] | All included studies are not RCT |
| 48 | Prognostic Value of Late Gadolinium Enhancement in Predicting Life-Threatening Arrhythmias in Heart Failure Patients With Implantable Cardioverter-Defibrillators: A Systematic Review and Meta-Analysis [48] | All included studies are not RCT |
| 49 | Wearable Sensors for Monitoring of Cigarette Smoking in Free-Living: A Systematic Review [49] | All included studies are not RCT |
| 50 | Systematic Review and Meta-Analysis of Clinical Outcome After Implantable Cardioverter-Defibrillator Therapy in Patients With Chagas Heart Disease [50] | All included studies are not RCT |
| 51 | Association Between Temporal Spatial Parameters and Overuse Injury History in Runners: A Systematic Review and Meta-analysis [51] | All included studies are not RCT |
| 52 | Predictors of Late Mortality in D-Transposition of the Great Arteries After Atrial Switch Repair: Systematic Review and Meta-Analysis [52] | All included studies are not RCT |
| 53 | Wearable sensors, cerebral palsy and gait assessment in everyday environments: is it a reality? - A systematic review [53] | All included studies are not RCT |
| 54 | A Systematic Review on the Value of Infrared Thermography in the Early Detection of Periprosthetic Joint Infections [54] | All included studies are not RCT |
| 55 | Analyzing the Use of Accelerometers as a Method of Early Diagnosis of Alterations in Balance in Elderly People: A Systematic Review [55] | All included studies are not RCT |
| 56 | Current State of Digital Biomarker Technologies for Real-Life, Home-Based Monitoring of Cognitive Function for Mild Cognitive Impairment to Mild Alzheimer Disease and Implications for Clinical Care: Systematic Review [56] | All included studies are not RCT |
| 57 | Subcutaneous implantable cardioverter-defibrillator in primary and secondary prevention of sudden cardiac death: A meta-analysis [57] | All included studies are not RCT |
| 58 | A Systematic Review and Implementation of IoT-Based Pervasive Sensor-Enabled Tracking System for Dementia Patients [58] | All included studies are not RCT |
| 59 | A systematic review of feasibility studies promoting the use of mobile technologies in clinical research [59] | All included studies are not RCT |
| 60 | Quantification of the validity and reliability of sprint performance metrics computed using inertial sensors: A systematic review [60] | All included studies are not RCT |
| 61 | Pooled Analysis of the Efficacy and Safety of Video Capsule Endoscopy in Patients with Implantable Cardiac Devices [61] | All included studies are not RCT |
| 62 | Toe Pressure in Predicting Diabetic Foot Ulcer Healing: A Systematic Review and Meta-analysis [62] | All included studies are not RCT |
| 63 | A Systematic Review of Wearable Sensors and IoT-Based Monitoring Applications for Older Adults - a Focus on Ageing Population and Independent Living [63] | All included studies are not RCT |
| 64 | Assessing Eating Behaviour Using Upper Limb Mounted Motion Sensors: A Systematic Review [64] | All included studies are not RCT |
| 65 | Accuracy and precision of transcutaneous carbon dioxide monitoring: a systematic review and meta-analysis [65] | Without health outcome |
| 66 | Assessment of Hypertension Using Clinical Electrocardiogram Features: A First-Ever Review [66] | Without health outcome |
| 67 | Physiological Measurements of Situation Awareness: A Systematic Review [67] | Without health outcome |
| 68 | The application of wearable smart sensors for monitoring the vital signs of patients in epidemics: a systematic literature review [68] | Without health outcome |
| 69 | The Use of Wearables in Clinical Trials During Cancer Treatment: Systematic Review [69] | Without health outcome |
| 70 | Mobile health applications for the detection of atrial fibrillation: a systematic review [70] | Without health outcome |
| 71 | Diagnostic and clinical values of non-cardiac ultrasound in COPD: A systematic review [71] | Without health outcome |
| 72 | Identification of Patient Perceptions That Can Affect the Uptake of Interventions Using Biometric Monitoring Devices: Systematic Review of Randomized Controlled Trials [72] | Without health outcome |
| 73 | Experiences of adults living with an implantable cardioverter defibrillator for cardiovascular disease: a systematic review of qualitative evidence [73] | Without health outcome |
| 74 | Better one or two? A systematic review of portable automated refractors [74] | Without health outcome |
| 75 | The Validity and Reliability of Wearable Microtechnology for Intermittent Team Sports: A Systematic Review [75] | Without health outcome |
| 76 | Wearable inertial sensors provide reliable biomarkers of disease severity in multiple sclerosis: A systematic review and meta-analysis [76] | Without health outcome |
| 77 | Can We Rely on Mobile Devices and Other Gadgets to Assess the Postural Balance of Healthy Individuals? A Systematic Review [77] | Without health outcome |
| 78 | Timing of device reimplantation and reinfection rates following cardiac implantable electronic device infection: a systematic review and meta-analysis [78] | Without health outcome |
| 79 | Measuring Physical Activity Using Triaxial Wrist Worn Polar Activity Trackers: A Systematic Review [79] | Without health outcome |
| 80 | Gait Kinematic Parameters in Parkinson's Disease: A Systematic Review [80] | Without health outcome |
| 81 | Systematic Review Looking at the Use of Technology to Measure Free-Living Symptom and Activity Outcomes in Parkinson's Disease in the Home or a Home-like Environment [81] | Without health outcome |
| 82 | Clinical Applications of Mobile Health Wearable-Based Sleep Monitoring: Systematic Review [82] | Without health outcome |
| 83 | Prevalence of obstructive sleep apnoea in acute coronary syndrome patients: systematic review and meta-analysis [83] | Without health outcome |
| 84 | A Systematic Review on the Use of Wearable Body Sensors for Health Monitoring: A Qualitative Synthesis [84] | Without health outcome |
| 85 | Comparison of arterial and venous implantable Doppler postoperative monitoring of free flaps: Systematic review and meta-analysis of diagnostic test accuracy [85] | Without health outcome |
| 86 | Automated Systems Based on Wearable Sensors for the Management of Parkinson's Disease at Home: A Systematic Review [86] | Without health outcome |
| 87 | Digital biomarkers from geolocation data in bipolar disorder and schizophrenia: a systematic review [87] | Without health outcome |
| 88 | The Cost-Effectiveness of Digital Health Interventions on the Management of Cardiovascular Diseases: Systematic Review [88] | Without health outcome |
| 89 | The role of wearable devices and objective gait analysis for the assessment and monitoring of patients with lumbar spinal stenosis: systematic review [89] | Without health outcome |
| 90 | Actigraphy for evaluation of mood disorders: A systematic review and meta-analysis [90] | Without health outcome |
| 91 | Cardiac arrhythmia detection outcomes among patients monitored with the Zio patch system: a systematic literature review [91] | Without health outcome |
| 92 | Mobile Apps to Quantify Aspects of Physical Activity: a Systematic Review on its Reliability and Validity [92] | Without health outcome |
| 93 | Efficacy of Portable Accelerometer-Based Navigation Devices versus Conventional Guides in Total Knee Arthroplasty: A Meta-analysis [93] | Without health outcome |
| 94 | Mobile Phone and Wearable Sensor-Based mHealth Approaches for Psychiatric Disorders and Symptoms: Systematic Review [94] | Without health outcome |
| 95 | Digital contact tracing technologies in epidemics: a rapid review [95] | Without health outcome |
| 96 | Role of (18)F-FDG PET/CT in the diagnosis of cardiovascular implantable electronic device infections: A meta-analysis [96] | Without health outcome |
| 97 | Detection of Near Falls Using Wearable Devices: A Systematic Review [97] | Without health outcome |
| 98 | Electromagnetic interference in cardiac electronic implants caused by novel electrical appliances emitting electromagnetic fields In the intermediate frequency range: a systematic review [98] | Without health outcome |
| 99 | How well do activity monitors estimate energy expenditure? A systematic review and meta-analysis of the validity of current technologies [99] | Without health outcome |
| 100 | Prognostic Value of Global Longitudinal Strain in Hypertrophic Cardiomyopathy: A Systematic Review of Existing Literature [100] | Without health outcome |
| 101 | Inertial sensors versus standard systems in gait analysis: a systematic review and meta-analysis [101] | Without health outcome |
| 102 | A Systematic Review of Technology-Driven Methodologies for Estimation of Energy Intake [102] | Without health outcome |
| 103 | Value of gait analysis for measuring disease severity using inertial sensors in patients with multiple sclerosis: protocol for a systematic review and meta-analysis [103] | Without health outcome |
| 104 | The Accuracy of Acquiring Heart Rate Variability from Portable Devices: A Systematic Review and Meta-Analysis [104] | Without health outcome |
| 105 | The role of wearables in spinal posture analysis: a systematic review [105] | Without health outcome |
| 106 | Assessing Gait in Parkinson's Disease Using Wearable Motion Sensors: A Systematic Review [106] | Without health outcome |
| 107 | The Key Factors in Physical Activity Type Detection Using Real-Life Data: A Systematic Review [107] | Without health outcome |
| 108 | Validity and Reliability of Wearable Sensors for Joint Angle Estimation: A Systematic Review [108] | Without health outcome |
| 109 | Patient acceptability of wearable vital sign monitoring technologies in the acute care setting: A systematic review [109] | Without health outcome |
| 110 | Augmented Reality in Medicine: Systematic and Bibliographic Review [110] | Without health outcome |
| 111 | Methodological considerations for kinematic analysis of upper limbs in healthy and poststroke adults Part II: a systematic review of motion capture systems and kinematic metrics [111] | Without health outcome |
| 112 | Accuracy of mHealth Devices for Atrial Fibrillation Screening: Systematic Review [112] | Without health outcome |
| 113 | Prediction of Ventricular Arrhythmias With Left Ventricular Mechanical Dispersion: A Systematic Review and Meta-Analysis [113] | Without health outcome |
| 114 | The use of Motor and Cognitive Dual-Task quantitative assessment on subjects with mild cognitive impairment: A systematic review [114] | Without health outcome |
| 115 | Screening test accuracy of portable devices that can be used to perform colposcopy for detecting CIN2+ in low- and middle-income countries: a systematic review and meta-analysis [115] | Without health outcome |
| 116 | Wearable Textile UHF-RFID Sensors: A Systematic Review [116] | Without health outcome |
| 117 | Short Versus Long Timing of Flushing of Totally Implantable Venous Access Devices When Not Used Routinely: A Systematic Review and Meta-analysis [117] | Without health outcome |
| 118 | Outcome measures and motion capture systems for assessing lower limb orthosis-based interventions after stroke: a systematic review [118] | Without health outcome |
| 119 | Use of Commercial Off-The-Shelf Devices for the Detection of Manual Gestures in Surgery: Systematic Literature Review [119] | Without health outcome |
| 120 | Acceptance and Use of Innovative Assistive Technologies among People with Cognitive Impairment and Their Caregivers: A Systematic Review [120] | Without health outcome |
| 121 | A systematic review of portable electronic technology for health education in resource-limited settings [121] | Without health outcome |
| 122 | Duration of Implantable Cardiac Monitoring and  Detection of Atrial Fibrillation in Ischemic Stroke  Patients: A Systematic Review and Meta-Analysis [122] | Without health outcome |
| 123 | Systematic review on the application of wearable inertial sensors to quantify everyday life motor activity in people with mobility impairments [123] | Without health outcome |
| 124 | The Effectiveness of Wearable Upper Limb Assistive Devices in Degenerative Neuromuscular Diseases: A Systematic Review and Meta-Analysis [124] | No digital biomarker-based |
| 125 | Technologies for Cognitive Training and Cognitive Rehabilitation for People With Mild Cognitive Impairment and Dementia. A Systematic Review [125] | No digital biomarker-based |
| 126 | The effectiveness of mHealth interventions on postpartum depression: A systematic review and meta-analysis [126] | No digital biomarker-based |
| 127 | The prognostic value of late gadolinium enhancement in myocarditis and clinically suspected myocarditis: systematic review and meta-analysis [127] | No digital biomarker-based |
| 128 | Perspectives of People Who Are Overweight and Obese on Using Wearable Technology for Weight Management: Systematic Review [128] | No digital biomarker-based |
| 129 | Non-implantable bone conduction device for hearing loss: a systematic review [129] | No digital biomarker-based |
| 130 | Accuracy of Portable Face-Scanning Devices for Obtaining Three-Dimensional Face Models: A Systematic Review and Meta-Analysis [130] | No digital biomarker-based |
| 131 | Implantable Subacromial Balloon Spacers in Patients With Massive Irreparable Rotator Cuff Tears: A Systematic Review of Clinical, Biomechanical, and Financial Implications [131] | No digital biomarker-based |
| 132 | Radiofrequency catheter ablation of ventricular tachycardia in ischemic heart disease in light of current practice: a systematic review and meta-analysis of randomized controlled trials [132] | No digital biomarker-based |
| 133 | Airway Clearance Using Suction Devices in Prehospital Combat Casualty Care: A Systematic Review [133] | No digital biomarker-based |
| 134 | Prolonging the flush-lock interval of totally implantable venous access ports in patients with cancer: A systematic review and meta-analysis [134] | No digital biomarker-based |
| 135 | The impact of continuous electrical microcurrent on acute and hard-to-heal wounds: a systematic review [135] | No digital biomarker-based |
| 136 | Performance Evaluation of Lower Limb Exoskeletons: A Systematic Review [136] | No digital biomarker-based |
| 137 | Adverse events related to electroacupuncture: a systematic review of single case studies and case series [137] | No digital biomarker-based |
| 138 | Association between CRT(D)/ICD and renal insufficiency: A systematic review and meta-analysis [138] | All included studies are not RCT |
| 139 | Wearable health technology to quantify the functional impact of peripheral neuropathy on mobility in Parkinson’s disease: A systematic review [139] | All included studies are not RCT |
| 140 | Biofeedback systems for gait rehabilitation of individuals with lower-limb amputation: A systematic review [140] | All included studies are not RCT |
| 141 | The Use of Activity Trackers in Interventions for Childhood Cancer Patients and Survivors: A Systematic Review [141] | All included studies are not RCT |
| 142 | Predefined vs data-guided training prescription based on autonomic nervous system variation: A systematic review [142] | All included studies are not RCT |
| 143 | Current reporting of usability and impact of mHealth interventions for substance use disorder: A systematic review [143] | All included studies are not RCT |
| 144 | Effects of real-time feedback on cardiopulmonary resuscitation quality on outcomes in adult patients with cardiac arrest: A systematic review and meta-analysis [144] | All included studies are not RCT |
| 145 | Effects of Motivational Interviewing and Wearable Fitness Trackers on Motivation and Physical Activity: A Systematic Review [145] | All included studies are not RCT |
| 146 | Diagnostic Performance of Prehospital Point-of-Care Troponin Tests to Rule out Acute Myocardial Infarction: A Systematic Review [146] | All included studies are not RCT |
| 147 | The role of an antibiotic envelope in the prevention of major cardiac implantable electronic device infections: A systematic review and meta-analysis [147] | All included studies are not RCT |
| 148 | Efficacy of antibacterial envelope in prevention of cardiovascular implantable electronic device infections in high-risk patients: A systematic review and meta-analysis [148] | All included studies are not RCT |
| 149 | Long-term weight management using wearable technology in overweight and obese adults: Systematic review [149] | All included studies are not RCT |
| 150 | Sensing technology to facilitate behavioral and psychological symptoms and to monitor treatment response in people with dementia: A systematic review [150] | All included studies are not RCT |
| 151 | Association of Exposures to Seated Postures With Immediate Increases in Back Pain: A Systematic Review of Studies With Objectively Measured Sitting Time [151] | All included studies are not RCT |
| 152 | Antibiotic envelope is associated with reduction in cardiac implantable electronic devices infections especially for high-power device—Systematic review and meta-analysis [152] | All included studies are not RCT |
| 153 | Implantable cardiac monitors to detect atrial fibrillation after cryptogenic stroke: A systematic review and economic evaluation [153] | All included studies are not RCT |
| 154 | Outcomes following implantable cardioverter–defibrillator generator replacement in adults: A systematic review [154] | All included studies are not RCT |
| 155 | Can Mobile Technology Improve Weight Loss in Overweight Adults? A Systematic Review [155] | All included studies are not RCT |
| 156 | Time trends in sudden cardiac death risk in heart failure patients with cardiac resynchronization therapy: A systematic review [156] | All included studies are not RCT |
| 157 | Device monitoring in heart failure management: Outcomes based on a systematic review and meta-analysis [157] | All included studies are not RCT |
| 158 | The Role of Technology in Adherence to Physical Activity Programs in Patients with Chronic Diseases Experiencing Fatigue: a Systematic Review [158] | All included studies are not RCT |
| 159 | Exercise Interventions in Patients with Implantable Cardioverter-Defibrillators and Cardiac Resynchronization Therapy: A SYSTEMATIC REVIEW and META-ANALYSIS [159] | All included studies are not RCT |
| 160 | Patient generated health data use in clinical practice: A systematic review [160] | All included studies are not RCT |
| 161 | Frailty, Implantable Cardioverter Defibrillators, and Mortality: a Systematic Review [161] | All included studies are not RCT |
| 162 | A Systematic Review of Electronic Health (eHealth) interventions to improve physical activity in patients with breast cancer [162] | All included studies are not RCT |
| 163 | Mobile health interventions to promote physical activity and reduce sedentary behaviour in the workplace: A systematic review [163] | All included studies are not RCT |
| 164 | Device infections in implantable cardioverter defibrillators versus permanent pacemakers: A systematic review and meta-analysis [164] | All included studies are not RCT |
| 165 | Data management and wearables in older adults: A systematic review [165] | All included studies are not RCT |
| 166 | Wearable Cardioverter-Defibrillator Therapy for the Prevention of Sudden Cardiac Death: A Systematic Review and Meta-Analysis [166] | All included studies are not RCT |
| 167 | Sleep Tracking: a Systematic Review of the Research Using Commercially Available Technology [167] | All included studies are not RCT |
| 168 | Association between atrial fibrillation and patient-important outcomes in heart failure patients with implantable cardioverter-defibrillators: A systematic review and meta-analysis [168] | All included studies are not RCT |
| 169 | Cardiac resynchronization therapy and outcomes in patients with left ventricular assist devices: a systematic review and meta-analysis [169] |  |

RCT: Randomized controlled trial

**References**

1. Thomas Craig KJ, Morgan LC, Chen CH, Michie S, Fusco N, Snowdon JL, Scheufele E, Gagliardi T, Sill S. Systematic review of context-aware digital behavior change interventions to improve health. Transl Behav Med 2021;11(5):1037–1048. PMID:33085767

2. Kanitsoraphan C, Rattanawong P, Mekraksakit P, Chongsathidkiet P, Riangwiwat T, Kanjanahattakij N, Vutthikraivit W, Klomjit S, Thavaraputta S. Baseline fragmented QRS is associated with increased all-cause mortality in heart failure with reduced ejection fraction: A systematic review and meta-analysis. Ann Noninvasive Electrocardiol [Internet] 2019 Mar;24(2):e12597. [doi: 10.1111/anec.12597]

3. Mahboub-Ahari A, Heidari F, Sadeghi-Ghyassi F, Asadi M. A systematic review of effectiveness and economic evaluation of Cardiohelp and portable devices for extracorporeal membrane oxygenation (ECMO). J Artif Organs [Internet] Springer Japan; 2019;22(1):6–13. PMID:30187234

4. Benz AP, Vamos M, Erath JW, Hohnloser SH. Cephalic vs. subclavian lead implantation in cardiac implantable electronic devices: A systematic review and meta-analysis. Europace 2019;21(1):121–129. PMID:30020452

5. El Moheb M, Nicolas J, Khamis AM, Iskandarani G, Akl EA, Refaat M. Implantable cardiac defibrillators for people with non-ischaemic cardiomyopathy. Cochrane Database Syst Rev 2018;2018(12). PMID:30537022

6. Miller J, Wells L, Nwulu U, Currow D, Johnson MJ, Skipworth RJE. Validated screening tools for the assessment of cachexia, sarcopenia, and malnutrition: A systematic review. Am J Clin Nutr Oxford University Press; 2018;108(6):1196–1208. PMID:30541096

7. Dong M, Fang B, Li J, Sun F, Liu H. Wearable sensing devices for upper limbs: A systematic review. Proc Inst Mech Eng Part H J Eng Med 2021;235(1):117–130. PMID:32885713

8. Follis S, Chen Z, Mishra S, Howe CL, Toosizadeh N, Dohm M. Comparison of wearable sensor to traditional methods in functional outcome measures: A systematic review. J Orthop Res 2021;39(10):2093–2102. PMID:33300119

9. Garg J, Shah K, Turagam MK, Tzou W, Gopinathannair R, Natale A, Lakkireddy D. Implantable cardioverter‐defibrillators in cardiac transplant recipients: A systematic review from the Electrophysiology Collaborative Consortium for Meta‐analysis—ELECTRAM investigators. Pacing Clin Electrophysiol [Internet] 2020 Dec 12;43(12):1529–1537. [doi: 10.1111/pace.14098]

10. Kamp NJ, Chery G, Kosinski AS, Desai MY, Wazni O, Schmidler GS, Patel M, Lopes RD, Morin DP, Al-Khatib SM. Risk stratification using late gadolinium enhancement on cardiac magnetic resonance imaging in patients with hypertrophic cardiomyopathy: A systematic review and meta-analysis. Prog Cardiovasc Dis [Internet] Elsevier Inc.; 2021;66:10–16. PMID:33171204

11. Menolotto M, Komaris DS, Tedesco S, O’flynn B, Walsh M. Motion capture technology in industrial applications: A systematic review. Sensors (Switzerland) 2020;20(19):1–25. PMID:33028042

12. Yuyun MF, Bonny A, Ng GA, Sliwa K, Kengne AP, Chin A, Mocumbi AO, Ngantcha M, Ajijola OA, Bukhman G. A systematic review of the spectrum of cardiac arrhythmias in Sub-Saharan Africa. Glob Heart 2020;15(1). PMID:32923331

13. Prasitlumkum N, Cheungpasitporn W, Chokesuwattanaskul A, Thangjui S, Thongprayoon C, Bathini T, Vallabhajosyula S, Kanitsoraphan C, Leesutipornchai T, Chokesuwattanaskul R. Diagnostic accuracy of smart gadgets/wearable devices in detecting atrial fibrillation: A systematic review and meta-analysis. Arch Cardiovasc Dis [Internet] Elsevier Masson SAS; 2021;114(1):4–16. PMID:32921618

14. Yue C, Yang F, Wang L, Li F, Chen Y. Association between fine particulate matter and atrial fibrillation in implantable cardioverter defibrillator patients: a systematic review and meta-analysis. J Interv Card Electrophysiol Journal of Interventional Cardiac Electrophysiology; 2020;59(3):595–601. PMID:32918184

15. Fuller D, Colwell E, Low J, Orychock K, Ann Tobin M, Simango B, Buote R, van Heerden D, Luan H, Cullen K, Slade L, Taylor NGA. Reliability and Validity of Commercially Available Wearable Devices for Measuring Steps, Energy Expenditure, and Heart Rate: Systematic Review. JMIR mHealth uHealth 2020;8(9):1–23. PMID:32897239

16. Niu Y, Chen Y, Li W, Xie R, Deng X. Electromagnetic interference effect of dental equipment on cardiac implantable electrical devices: A systematic review. PACE - Pacing Clin Electrophysiol 2020;43(12):1588–1598. PMID:32852847

17. Kohl SH, Mehler DMA, Lührs M, Thibault RT, Konrad K, Sorger B. The Potential of Functional Near-Infrared Spectroscopy-Based Neurofeedback—A Systematic Review and Recommendations for Best Practice. Front Neurosci 2020;14(July). [doi: 10.3389/fnins.2020.00594]

18. Benoit J, Onyeaka H, Keshavan M, Torous J. Systematic Review of Digital Phenotyping and Machine Learning in Psychosis Spectrum Illnesses. Harv Rev Psychiatry 2020;28(5):296–304. PMID:32796192

19. van Egmond K, Wright CJC, Livingston M, Kuntsche E. Wearable Transdermal Alcohol Monitors: A Systematic Review of Detection Validity, and Relationship Between Transdermal and Breath Alcohol Concentration and Influencing Factors. Alcohol Clin Exp Res 2020;44(10):1918–1932. PMID:32767791

20. Cajamarca G, Herskovic V, Rossel PO. Enabling older adults’ health self-management through self-report and visualization—a systematic literature review†. Sensors (Switzerland) 2020;20(15):1–16. PMID:32759801

21. Villarreal V, Berbey-Alvarez A. Evaluation of mhealth applications related to cardiovascular diseases: A systematic review. Acta Inform Medica 2020;28(2):130–137. [doi: 10.5455/aim.2020.28.130-137]

22. Gonzalez-Jaramillo V, Sobanski P, Calvache JA, Arenas-Ochoa LF, Franco OH, Hunziker L, Eychmüller S, Maessen M. Unmet device reprogramming needs at the end of life among patients with implantable cardioverter defibrillator: A systematic review and meta-analysis. Palliat Med 2020;34(8):1019–1029. PMID:32588755

23. Zhang Y, Weaver RG, Armstrong B, Burkart S, Zhang S, Beets MW. Validity of Wrist-Worn photoplethysmography devices to measure heart rate: A systematic review and meta-analysis. J Sports Sci [Internet] Routledge; 2020;38(17):2021–2034. PMID:32552580

24. Wang TKM, Sánchez-Nadales A, Igbinomwanhia E, Cremer P, Griffin B, Xu B. Diagnosis of Infective Endocarditis by Subtype Using 18F-Fluorodeoxyglucose Positron Emission Tomography/Computed Tomography: A Contemporary Meta-Analysis. Circ Cardiovasc Imaging 2020;(June):1–11. PMID:32507019

25. Chew DS, Rennert-May E, Spackman E, Mark DB, Exner D V. Cost-effectiveness of extended electrocardiogram monitoring for atrial fibrillation after stroke a systematic review. Stroke 2020;51(7):2244–2248. PMID:32498661

26. Leenen JPL, Leerentveld C, van Dijk JD, van Westreenen HL, Schoonhoven L, Patijn GA. Current evidence for continuous vital signs monitoring by wearable wireless devices in hospitalized adults: Systematic review. J Med Internet Res 2020;22(6). PMID:32469323

27. Butz AM, Christopher S. von Bartheld JB and SH-H. 乳鼠心肌提取 HHS Public Access. Physiol Behav 2017;176(12):139–148. [doi: 10.1016/j.jad.2020.03.096.Objective]

28. Kobsar D, Charlton JM, Tse CTF, Esculier JF, Graffos A, Krowchuk NM, Thatcher D, Hunt MA. Validity and reliability of wearable inertial sensors in healthy adult walking: A systematic review and meta-analysis. J Neuroeng Rehabil Journal of NeuroEngineering and Rehabilitation; 2020;17(1):1–21. PMID:32393301

29. Halawa A, Woldu HG, Kacey KG, Alpert MA. Effect of ICD implantation on cardiovascular outcomes in patients with cardiac amyloidosis: A systematic review and meta-anaylsis. J Cardiovasc Electrophysiol 2020;31(7):1749–1758. PMID:32391952

30. Storm FA, Cesareo A, Reni G, Biffi E. Wearable inertial sensors to assess gait during the 6-minute walk test: A systematic review. Sensors (Switzerland) 2020;20(9). PMID:32384806

31. Black-Maier E, Lewis RK, Barnett AS, Pokorney SD, Sun AY, Koontz JI, Daubert JP, Piccini JP. Subcutaneous implantable cardioverter-defibrillator troubleshooting in patients with a left ventricular assist device: A case series and systematic review. Hear Rhythm [Internet] Heart Rhythm Society; 2020;17(9):1536–1544. PMID:32304733

32. Sammani A, Kayvanpour E, Bosman LP, Sedaghat-Hamedani F, Proctor T, Gi WT, Broezel A, Jensen K, Katus HA, te Riele ASJM, Meder B, Asselbergs FW. Predicting sustained ventricular arrhythmias in dilated cardiomyopathy: a meta-analysis and systematic review. ESC Hear Fail 2020;7(4):1430–1441. PMID:32285648

33. Zhang Z, Brazil J, Ozkaynak M, Desanto K. Evaluative Research of Technologies for Prehospital Communication and Coordination: a Systematic Review. J Med Syst 2020;44(5). PMID:32246206

34. Vinetti G, Lopomo NF, Taboni A, Fagoni N, Ferretti G. The current use of wearable sensors to enhance safety and performance in breath-hold diving: A systematic review. Diving Hyperb Med 2020;50(1):54–65. PMID:32187619

35. Campbell E, Phinyomark A, Scheme E. Current trends and confounding factors in myoelectric control: Limb position and contraction intensity. Sensors (Switzerland) 2020;20(6):1–44. PMID:32183215

36. Oshvandi K, Khatiban M, Ghanei Gheshlagh R, Razavi M. The prevalence of depression in patients living with implantable cardioverter defibrillator: a systematic review and meta-analysis. Ir J Med Sci Irish Journal of Medical Science (1971 -); 2020;189(4):1243–1252. PMID:32172313

37. Davoudi A, Manini TM, Bihorac A, Rashidi P. Role of Wearable Accelerometer Devices in Delirium Studies. Crit Care Explor 2019;1(9):e0027. [doi: 10.1097/cce.0000000000000027]

38. Rattanawong P, Kewcharoen J, Techorueangwiwat C, Kanitsoraphan C, Mekritthikrai R, Prasitlumkum N, Puttapiban P, Mekraksakit P, Vutthikraivit W, Sorajja D. Wide QRS complex and the risk of major arrhythmic events in Brugada syndrome patients: A systematic review and meta-analysis. J Arrhythmia 2020;36(1):143–152. [doi: 10.1002/joa3.12290]

39. Amirfaiz S, Shahril MR. Objectively Measured Physical Activity, Sedentary Behavior, and Metabolic Syndrome in Adults: Systematic Review of Observational Evidence. Metab Syndr Relat Disord 2019;17(1):1–21. PMID:30272527

40. Munawar DA, Chan JEZ, Emami M, Kadhim K, Khokhar K, O’shea C, Iwai S, Pitman B, Linz D, Munawar M, Roberts-Thomson K, Young GD, Mahajan R, Sanders P, Lau DH. Magnetic resonance imaging in non-conditional pacemakers and implantable cardioverter-defibrillators: A systematic review and meta-analysis. Europace 2020;22(2):288–298. PMID:31995177

41. Millán CA, Girón NA, Lopez DM. Analysis of relevant features from photoplethysmographic signals for atrial fibrillation classification. Int J Environ Res Public Health 2020;17(2). PMID:31941071

42. Scott H, Lack L, Lovato N. A systematic review of the accuracy of sleep wearable devices for estimating sleep onset. Sleep Med Rev [Internet] Elsevier Ltd; 2020;49:101227. PMID:31901524

43. Haghayegh S, Khoshnevis S, Smolensky MH, Diller KR, Castriotta RJ. Accuracy of wristband fitbit models in assessing sleep: Systematic review and meta-analysis. J Med Internet Res 2019;21(11). PMID:31778122

44. Berthelot M, Ashcroft J, Boshier P, Hunter J, Henry FP, Lo B, Yang GZ, Leff D. Use of Near-infrared Spectroscopy and Implantable Doppler for Postoperative Monitoring of Free Tissue Transfer for Breast Reconstruction: A Systematic Review and Meta-analysis. Plast Reconstr Surg - Glob Open 2019;7(10):E2437. [doi: 10.1097/GOX.0000000000002437]

45. Zhong R, Rau PLP. Are cost-effective technologies feasible to measure gait in older adults? A systematic review of evidence-based literature. Arch Gerontol Geriatr [Internet] Elsevier; 2020;87(October 2019):103970. PMID:31743825

46. Smagula SF, Gujral S, Capps CS, Krafty RT. A systematic review of evidence for a role of rest-activity rhythms in dementia. Front Psychiatry 2019;10(OCT):1–7. [doi: 10.3389/fpsyt.2019.00778]

47. Carnevale A, Longo UG, Schena E, Massaroni C, Lo Presti D, Berton A, Candela V, Denaro V. 肩关节运动评估的可穿戴系统:系统综述. BMC Musculoskelet Disord BMC Musculoskeletal Disorders; 2019;20(1).

48. Yue T, Chen B, Wu L, Xu J, Pu J. Prognostic Value of Late Gadolinium Enhancement in Predicting Life‐Threatening Arrhythmias in Heart Failure Patients With Implantable Cardioverter‐Defibrillators: A Systematic Review and Meta‐Analysis. J Magn Reson Imaging [Internet] 2020 May 11;51(5):1422–1439. [doi: 10.1002/jmri.26982]

49. Imtiaz MH, Ramos-Garcia RI, Wattal S, Tiffany S, Sazonov E. Wearable sensors for monitoring of cigarette smoking in free-living: A systematic review. Sensors (Switzerland) 2019;19(21). PMID:31661856

50. Rassi FM, Minohara L, Rassi A, Correia LCL, Marin-Neto JA, da Silva Menezes A. Systematic Review and Meta-Analysis of Clinical Outcome After Implantable Cardioverter-Defibrillator Therapy in Patients With Chagas Heart Disease. JACC Clin Electrophysiol 2019;5(10):1213–1223. PMID:31648747

51. Brindle RA, Taylor JB, Rajek C, Weisbrod A, Ford KR. Association Between Temporal Spatial Parameters and Overuse Injury History in Runners: A Systematic Review and Meta-analysis. Sport Med [Internet] Springer International Publishing; 2020;50(2):331–342. PMID:31643019

52. Venkatesh P, Evans AT, Maw AM, Pashun RA, Patel A, Kim L, Feldman D, Minutello R, Wong SC, Stribling JC, LaPar D, Holzer R, Ginns J, Bacha E, Singh HS. Predictors of Late Mortality in D-Transposition of the Great Arteries After Atrial Switch Repair: Systematic Review and Meta-Analysis. J Am Heart Assoc 2019;8(21). PMID:31642369

53. Rozin Kleiner AF, Belgamo A, Pagnussat AS, Costa e Silva A de A, Sforza C, Rocha NACF. Wearable sensors, cerebral palsy and gait assessment in everyday environments: Is it a reality? - A systematic review. Funct Neurol 2019;34(2):85–91. PMID:31556388

54. Scheidt S, Rüwald J, Schildberg FA, Mahlein AK, Seuser A, Wirtz DC, Jacobs C. A Systematic Review on the Value of Infrared Thermography in the Early Detection of Periprosthetic Joint Infections. Z Orthop Unfall 2020;158(4):397–405. PMID:31525794

55. Leirós-Rodríguez R, García-Soidán JL, Romo-Pérez V. Analyzing the use of accelerometers as a method of early diagnosis of alterations in balance in elderly people: A systematic review. Sensors (Switzerland) 2019;19(18). PMID:31505828

56. Piau A, Wild K, Mattek N, Kaye J. Current state of digital biomarker technologies for real-life, home-based monitoring of cognitive function for mild cognitive impairment to mild Alzheimer disease and implications for clinical care: Systematic review. J Med Internet Res 2019;21(8). PMID:31471958

57. León Salas B, Trujillo-Martín MM, García García J, Ramallo Fariña Y, García Quintana A, Quirós López R, Serrano-Aguilar P. Subcutaneous implantable cardioverter-defibrillator in primary and secondary prevention of sudden cardiac death: A meta-analysis. PACE - Pacing Clin Electrophysiol 2019;42(9):1253–1268. PMID:31396970

58. Ray PP, Dash D, De D. A Systematic Review and Implementation of IoT-Based Pervasive Sensor-Enabled Tracking System for Dementia Patients. J Med Syst Journal of Medical Systems; 2019;43(9). PMID:31317281

59. Bakker JP, Goldsack JC, Clarke M, Coravos A, Geoghegan C, Godfrey A, Heasley MG, Karlin DR, Manta C, Peterson B, Ramirez E, Sheth N, Bruno A, Bullis E, Wareham K, Zimmerman N, Forrest A, Wood WA. A systematic review of feasibility studies promoting the use of mobile technologies in clinical research. npj Digit Med [Internet] Springer US; 2019;2(1). [doi: 10.1038/s41746-019-0125-x]

60. Macadam P, Cronin J, Neville J, Diewald S. Quantification of the validity and reliability of sprint performance metrics computed using inertial sensors: A systematic review. Gait Posture [Internet] Elsevier; 2019;73(July):26–38. PMID:31299501

61. Tabet R, Nassani N, Karam B, Shammaa Y, Akhrass P, Deeb L. Pooled Analysis of the Efficacy and Safety of Video Capsule Endoscopy in Patients with Implantable Cardiac Devices. Can J Gastroenterol Hepatol Hindawi; 2019;2019:2–7. PMID:31236386

62. Tay WL, Lo ZJ, Hong Q, Yong E, Chandrasekar S, Tan GWL. Toe Pressure in Predicting Diabetic Foot Ulcer Healing: A Systematic Review and Meta-analysis. Ann Vasc Surg [Internet] Elsevier Inc.; 2019;60:371–378. PMID:31220591

63. Baig MM, Afifi S, GholamHosseini H, Mirza F. A Systematic Review of Wearable Sensors and IoT-Based Monitoring Applications for Older Adults – a Focus on Ageing Population and Independent Living. J Med Syst 2019;43(8):10916. PMID:31203472

64. Heydarian H, Adam M, Burrows T, Collins C, Rollo ME. Assessing eating behaviour using upper limb mounted motion sensors: A systematic review. Nutrients 2019;11(5). PMID:31137677

65. Conway A, Tipton E, Liu WH, Conway Z, Soalheira K, Sutherland J, Fingleton J. Accuracy and precision of transcutaneous carbon dioxide monitoring: A systematic review and meta-analysis. Thorax 2019;74(2):157–163. PMID:30209079

66. Bird K, Chan G, Lu H, Greeff H, Allen J, Abbott D, Menon C, Lovell NH, Howard N, Chan WS, Fletcher RR, Alian A, Ward R, Elgendi M. Assessment of Hypertension Using Clinical Electrocardiogram Features: A First-Ever Review. Front Med 2020;7(December):1–17. PMID:33344473

67. Zhang T, Yang J, Liang N, Pitts BJ, Prakah-Asante KO, Curry R, Duerstock BS, Wachs JP, Yu D. Physiological Measurements of Situation Awareness: A Systematic Review. Hum Factors 2020;(3):1–22. PMID:33241945

68. Mohammadzadeh N, Gholamzadeh M, Saeedi S, Rezayi S. The application of wearable smart sensors for monitoring the vital signs of patients in epidemics: a systematic literature review. J Ambient Intell Humaniz Comput [Internet] Springer Berlin Heidelberg; 2020;(0123456789). [doi: 10.1007/s12652-020-02656-x]

69. Beauchamp UL, Pappot H, Holländer-Mieritz C. The use of wearables in clinical trials during cancer treatment: Systematic review. JMIR mHealth uHealth 2020;8(11):1–15. PMID:33174852

70. Lopez Perales CR, Van Spall HGC, Maeda S, Jimenez A, Laţcu DG, Milman A, Kirakoya-Samadoulougou F, Mamas MA, Muser D, Casado Arroyo R. Mobile health applications for the detection of atrial fibrillation: A systematic review. Europace 2021;23(1):11–28. PMID:33043358

71. Alqahtani JS, Oyelade T, Sreedharan J, Aldhahir AM, Alghamdi SM, Alrajeh AM, Alqahtani AS, Alsulayyim A, Aldabayan YS, Alobaidi NY, AlAhmari MD. Diagnostic and clinical values of non-cardiac ultrasound in COPD: A systematic review. BMJ open Respir Res 2020;7(1):1–8. PMID:32978244

72. Perlmutter A, Benchoufi M, Ravaud P, Tran VT. Identification of patient perceptions that can affect the uptake of interventions using biometric monitoring devices: Systematic review of randomized controlled trials. J Med Internet Res 2020;22(9). PMID:32915153

73. Pike A, Dobbin-Williams K, Swab M. Experiences of adults living with an implantable cardioverter defibrillator for cardiovascular disease: a systematic review of qualitative evidence. JBI Evid Synth [Internet] 2020 Nov;18(11):2231–2301. [doi: 10.11124/JBISRIR-D-19-00239]

74. Samanta A, Shetty A, Nelson PC. Better one or two? A systematic review of portable automated refractors. J Telemed Telecare 2020; [doi: 10.1177/1357633X20940140]

75. Crang ZL, Duthie G, Cole MH, Weakley J, Hewitt A, Johnston RD. The Validity and Reliability of Wearable Microtechnology for Intermittent Team Sports: A Systematic Review. Sport Med [Internet] Springer International Publishing; 2021;51(3):549–565. [doi: 10.1007/s40279-020-01399-1]

76. Vienne-Jumeau A, Quijoux F, Vidal PP, Ricard D. Wearable inertial sensors provide reliable biomarkers of disease severity in multiple sclerosis: A systematic review and meta-analysis. Ann Phys Rehabil Med 2020;63(2):138–147. PMID:31421274

77. Pinho AS, Salazar AP, Hennig EM, Spessato BC, Domingo A, Pagnussat AS. Can we rely on mobile devices and other gadgets to assess the postural balance of healthy individuals? A systematic review. Sensors (Switzerland) 2019;19(13). PMID:31284455

78. Chew D, Somayaji R, Conly J, Exner D, Rennert-May E. Timing of device reimplantation and reinfection rates following cardiac implantable electronic device infection: A systematic review and meta-analysis. BMJ Open 2019;9(9):1–9. PMID:31481556

79. Henriksen A, Johansson J, Hartvigsen G, Grimsgaard S, Hopstock L. Measuring physical activity using triaxial wrist worn polar activity trackers: A systematic review. Int J Exerc Sci 2020;13(4):438–454. PMID:32509122

80. Bouça-Machado R, Jalles C, Guerreiro D, Pona-Ferreira F, Branco Di, Guerreiro T, Matias R, Ferreira JJ. Gait Kinematic Parameters in Parkinson’s Disease: A Systematic Review. J Parkinsons Dis 2020;10(3):843–853. PMID:32417796

81. Morgan C, Rolinski M, McNaney R, Jones B, Rochester L, Maetzler W, Craddock I, Whone AL. Systematic Review Looking at the Use of Technology to Measure Free-Living Symptom and Activity Outcomes in Parkinson’s Disease in the Home or a Home-like Environment. J Parkinsons Dis 2020;10(2):429–454. PMID:32250314

82. Guillodo E, Lemey C, Simonnet M, Walter M, Baca-García E, Masetti V, Moga S, Larsen M, Ropars J, Berrouiguet S. Clinical applications of mobile health wearable–based sleep monitoring: Systematic review. JMIR mHealth uHealth 2020;8(4):1–10. [doi: 10.2196/10733]

83. Le Grande MR, Beauchamp A, Driscoll A, Jackson AC. Prevalence of obstructive sleep apnoea in acute coronary syndrome patients: Systematic review and meta-analysis. BMC Cardiovasc Disord BMC Cardiovascular Disorders; 2020;20(1):1–11. PMID:32209053

84. Kristoffersson A, Lindén M. A systematic review on the use of wearable body sensors for health monitoring: A qualitative synthesis. Sensors (Switzerland) 2020;20(5). PMID:32182907

85. Klifto KM, Milek D, Gurno CF, Seal SM, Hultman CS, Rosson GD, Cooney DS. Comparison of arterial and venous implantable Doppler postoperative monitoring of free flaps: Systematic review and meta-analysis of diagnostic test accuracy. Microsurgery 2020;40(4):501–511. PMID:32031735

86. Rovini E, Maremmani C, Cavallo F. Automated Systems Based on Wearable Sensors for the Management of Parkinson’s Disease at Home: A Systematic Review. Telemed e-Health 2019;25(3):167–183. PMID:29969384

87. Fraccaro P, Beukenhorst A, Sperrin M, Harper S, Palmier-Claus J, Lewis S, Van Der Veer SN, Peek N. Digital biomarkers from geolocation data in bipolar disorder and schizophrenia: A systematic review. J Am Med Informatics Assoc 2019;26(11):1412–1420. PMID:31260049

88. Jiang X, Ming WK, You JHS. The cost-effectiveness of digital health interventions on the management of cardiovascular diseases: Systematic review. J Med Internet Res 2019;21(6):1–11. PMID:31210136

89. Chakravorty A, Mobbs RJ, Anderson DB, Rooke K, Phan K, Yoong N, Maharaj M, Choy WJ. The role of wearable devices and objective gait analysis for the assessment and monitoring of patients with lumbar spinal stenosis: Systematic review. BMC Musculoskelet Disord BMC Musculoskeletal Disorders; 2019;20(1):1–9. PMID:31202276

90. Tazawa Y, Wada M, Mitsukura Y, Takamiya A, Kitazawa M, Yoshimura M, Mimura M, Kishimoto T. Actigraphy for evaluation of mood disorders: A systematic review and meta-analysis. J Affect Disord [Internet] Elsevier B.V.; 2019;253(February):257–269. PMID:31060012

91. Yenikomshian M, Jarvis J, Patton C, Yee C, Mortimer R, Birnbaum H, Topash M. Cardiac arrhythmia detection outcomes among patients monitored with the Zio patch system: a systematic literature review. Curr Med Res Opin [Internet] 2019 Oct 3;35(10):1659–1670. [doi: 10.1080/03007995.2019.1610370]

92. Silva AG, Simões P, Queirós A, Rodrigues M, Rocha NP. Mobile Apps to Quantify Aspects of Physical Activity: a Systematic Review on its Reliability and Validity. J Med Syst 2020;44(2). PMID:31915935

93. Sun H, Li S, Wang K, Wu G, Zhou J, Sun X. Efficacy of Portable Accelerometer-Based Navigation Devices versus Conventional Guides in Total Knee Arthroplasty: A Meta-analysis. J Knee Surg 2020;33(7):691–703. PMID:30959545

94. Seppälä J, De Vita I, Jämsä T, Miettunen J, Isohanni M, Rubinstein K, Feldman Y, Grasa E, Corripio I, Berdun J, D’Amico E, Bulgheroni M. Mobile phone and wearable sensor-based mhealth approaches for psychiatric disorders and symptoms: Systematic review. JMIR Ment Heal 2019;6(2):1–14. PMID:30785404

95. Anglemyer A. Digital contact tracing technologies in epiDemics: A rapid review. Saudi Med J 2020;41(9):1028. PMID:33502000

96. Mahmood M, Kendi AT, Farid S, Ajmal S, Johnson GB, Baddour LM, Chareonthaitawee P, Friedman PA, Sohail MR. Role of 18F-FDG PET/CT in the diagnosis of cardiovascular implantable electronic device infections: A meta-analysis. J Nucl Cardiol Springer US; 2019;26(3):958–970. PMID:28913626

97. Pang I, Okubo Y, Sturnieks D, Lord SR, Brodie MA. Detection of Near Falls Using Wearable Devices: A Systematic Review. J Geriatr Phys Ther 2019;42(1):48–56. PMID:29384813

98. Driessen S, Napp A, Schmiedchen K, Kraus T, Stunder D. Electromagnetic interference in cardiac electronic implants caused by novel electrical appliances emitting electromagnetic fields in the intermediate frequency range: A systematic review. Europace 2019;21(2):219–229. PMID:29992289

99. O’Driscoll R, Turicchi J, Beaulieu K, Scott S, Matu J, Deighton K, Finlayson G, Stubbs J. How well do activity monitors estimate energy expenditure? A systematic review and meta-analysis of the validity of current technologies. Br J Sports Med 2020;54(6):332–340. PMID:30194221

100. Tower-Rader A, Mohananey D, To A, Lever HM, Popovic ZB, Desai MY. Prognostic Value of Global Longitudinal Strain in Hypertrophic Cardiomyopathy: A Systematic Review of Existing Literature. JACC Cardiovasc Imaging 2019;12(10):1930–1942. PMID:30219395

101. Petraglia F, Scarcella L, Pedrazzi G, Brancato L, Puers R, Costantino C. Inertial sensors versus standard systems in gait analysis: A systematic review and meta-analysis. Eur J Phys Rehabil Med 2019;55(2):265–280. PMID:30311493

102. Doulah A, Mccrory MA, Higgins JA, Sazonov E. A Systematic Review of Technology-Driven Methodologies for Estimation of Energy Intake. IEEE Access IEEE; 2019;7(January):49653–49668. [doi: 10.1109/ACCESS.2019.2910308]

103. Vienne-Jumeau A, Quijoux F, Vidal PP, Ricard D. Value of gait analysis for measuring disease severity using inertial sensors in patients with multiple sclerosis: Protocol for a systematic review and meta-analysis. Syst Rev Systematic Reviews; 2019;8(1):1–5. PMID:30621765

104. Dobbs WC, Fedewa M V., MacDonald H V., Holmes CJ, Cicone ZS, Plews DJ, Esco MR. The Accuracy of Acquiring Heart Rate Variability from Portable Devices: A Systematic Review and Meta-Analysis. Sport Med [Internet] Springer International Publishing; 2019;49(3):417–435. PMID:30706234

105. Simpson L, Maharaj MM, Mobbs RJ. The role of wearables in spinal posture analysis: A systematic review. BMC Musculoskelet Disord BMC Musculoskeletal Disorders; 2019;20(1):1–14. PMID:30736775

106. Brognara L, Palumbo P, Grimm B, Palmerini L. Assessing Gait in Parkinson’s Disease Using Wearable Motion Sensors: A Systematic Review. Diseases 2019;7(1):18. [doi: 10.3390/diseases7010018]

107. Allahbakhshi H, Hinrichs T, Huang H, Weibel R. The key factors in physical activity type detection using real-life data: A systematic review. Front Physiol 2019;10(FEB):1–20. [doi: 10.3389/fphys.2019.00075]

108. Poitras I, Dupuis F, Bielmann M, Campeau-Lecours A, Mercier C, Bouyer LJ, Roy JS. Validity and reliability ofwearable sensors for joint angle estimation: A systematic review. Sensors (Switzerland) 2019;19(7):1–17. PMID:30935116

109. Sprogis SK, Currey J, Considine J. Patient acceptability of wearable vital sign monitoring technologies in the acute care setting: A systematic review. J Clin Nurs 2019;28(15–16):2732–2744. PMID:31017338

110. Eckert M, Volmerg JS, Friedrich CM. Augmented reality in medicine: Systematic and bibliographic review. JMIR mHealth uHealth 2019;7(4). [doi: 10.2196/10967]

111. Mesquita IA, Fonseca PFP da, Pinheiro ARV, Velhote Correia MFP, Silva CIC da. Methodological considerations for kinematic analysis of upper limbs in healthy and poststroke adults Part II: a systematic review of motion capture systems and kinematic metrics. Top Stroke Rehabil [Internet] Taylor & Francis; 2019;26(6):464–472. PMID:31064281

112. Giebel GD, Gissel C. Accuracy of mhealth devices for atrial fibrillation screening: Systematic review. JMIR mHealth uHealth 2019;7(6):1–13. PMID:31199337

113. Kawakami H, Nerlekar N, Haugaa KH, Edvardsen T, Marwick TH. Prediction of Ventricular Arrhythmias With Left Ventricular Mechanical Dispersion: A Systematic Review and Meta-Analysis. JACC Cardiovasc Imaging 2020;13(2P2):562–572. PMID:31202762

114. Mancioppi G, Fiorini L, Rovini E, Cavallo F. The use of Motor and Cognitive Dual-Task quantitative assessment on subjects with mild cognitive impairment: A systematic review. Mech Ageing Dev [Internet] Elsevier B.V.; 2021;193(August 2020):111393. PMID:33188785

115. Taghavi K, Rohner E, Basu P, Low N, Rutjes A, Bohlius J. Screening test accuracy of portable devices that can be used to perform colposcopy for detecting CIN2+ in low- and middle-income countries: a systematic review and meta-analysis. BMC Womens Health [Internet] BioMed Central; 2020;20(1):1–13. PMID:33198721

116. Luo C, Gil I, Fernández-García R. Wearable textile UHF-RFID sensors: A systematic review. Materials (Basel) 2020;13(15). [doi: 10.3390/ma13153292]

117. Clari M, Spoto M, Franceschi G, Acuto M, Tonella S, Caristia S, Buratti G, Gaboardi S, Rasero L, Campagna S, Busca E, Dal Molin A. Short Versus Long Timing of Flushing of Totally Implantable Venous Access Devices When Not Used Routinely: A Systematic Review and Meta-analysis. Cancer Nurs 2021;44(3):205–213. PMID:32384421

118. Figueiredo J, Moreno JC, Matias AC, Pereira F, Santos CP. Outcome measures and motion capture systems for assessing lower limb orthosis-based interventions after stroke: a systematic review. Disabil Rehabil Assist Technol [Internet] Taylor & Francis; 2021;16(6):674–683. PMID:31815572

119. Alvarez-Lopez F, Maina MF, Saigí-Rubió F. Use of commercial off-the-shelf devices for the detection of manual gestures in surgery: Systematic literature review. J Med Internet Res 2019;21(5):1–26. PMID:31066679

120. Thordardottir B, Malmgren Fänge A, Lethin C, Rodriguez Gatta D, Chiatti C. Acceptance and Use of Innovative Assistive Technologies among People with Cognitive Impairment and Their Caregivers: A Systematic Review. Biomed Res Int 2019;2019. PMID:30956989

121. McHenry MS, Fischer LJ, Chun Y, Vreeman RC. A systematic review of portable electronic technology for health education in resource-limited settings. Glob Health Promot 2019;26(2):70–81. PMID:28832243

122. Tsivgoulis G, Katsanos AH, Köhrmann M, Caso V, Perren F, Palaiodimou L, Deftereos S, Giannopoulos S, Ellul J, Krogias C, Mavridis D, Triantafyllou S, Alexandrov AW, Schellinger PD, Alexandrov A V. Duration of implantable cardiac monitoring and detection of atrial fibrillation in ischemic stroke patients: A systematic review and meta-analysis. J Stroke 2019;21(3):302–311. [doi: 10.5853/jos.2019.01067]

123. Rast FM, Labruyère R. Systematic review on the application of wearable inertial sensors to quantify everyday life motor activity in people with mobility impairments. J Neuroeng Rehabil [Internet] BioMed Central; 2020;17(1). PMID:33148315

124. Gandolla M, Antonietti A, Longatelli V, Pedrocchi A. The Effectiveness of Wearable Upper Limb Assistive Devices in Degenerative Neuromuscular Diseases: A Systematic Review and Meta-Analysis. Front Bioeng Biotechnol 2020;7(January):1–16. [doi: 10.3389/fbioe.2019.00450]

125. Irazoki E, Contreras-Somoza LM, Toribio-Guzmán JM, Jenaro-Río C, Van Der Roest H, Franco-Martín MA. Technologies for cognitive training and cognitive rehabilitation for people with mild cognitive impairment and dementia. A systematic review. Front Psychol 2020;11(April). PMID:32373018

126. Zhou C, Hu H, Wang C, Zhu Z, Feng G, Xue J, Yang Z. The effectiveness of mHealth interventions on postpartum depression: A systematic review and meta-analysis. J Telemed Telecare 2022;28(2):83–95. PMID:32306847

127. Yang F, Wang J, Li W, Xu Y, Wan K, Zeng R, Chen Y. The prognostic value of late gadolinium enhancement in myocarditis and clinically suspected myocarditis: systematic review and meta-analysis. Eur Radiol European Radiology; 2020;30(5):2616–2626. PMID:32040731

128. Hu R, van Velthoven MH, Meinert E. Perspectives of people who are overweight and obese on using wearable technology for weight management: Systematic review. JMIR mHealth uHealth 2020;8(1). PMID:31929104

129. A Moffa , L Giorgi, M Cassano , V Rinaldi , A Natalizia , F Bressi , E Guglielmelli MC. Non-implantable bone conduction device for hearing loss: a systematic review. J Biol Regul Homeost Agents 2020;

130. Mai HN, Kim J, Choi YH, Lee DH. Accuracy of portable face-scanning devices for obtaining three-dimensional face models: A systematic review and meta-analysis. Int J Environ Res Public Health 2021;18(1):1–15. PMID:33375533

131. Johns WL, Ailaney N, Lacy K, Golladay GJ, Vanderbeck J, Kalore N V. Implantable Subacromial Balloon Spacers in Patients With Massive Irreparable Rotator Cuff Tears: A Systematic Review of Clinical, Biomechanical, and Financial Implications. Arthrosc Sport Med Rehabil [Internet] Arthroscopy Association of North America; 2020;2(6):e855–e872. [doi: 10.1016/j.asmr.2020.06.011]

132. Lima da Silva G, Nunes-Ferreira A, Cortez-Dias N, de Sousa J, J. Pinto F, Caldeira D. Radiofrequency catheter ablation of ventricular tachycardia in ischemic heart disease in light of current practice: a systematic review and meta-analysis of randomized controlled trials. J Interv Card Electrophysiol Journal of Interventional Cardiac Electrophysiology; 2020;59(3):603–616. PMID:32948937

133. Jain P, Akhter F, Schoppe A, Hood RL, De Lorenzo RA. Airway Clearance Using Suction Devices in Prehospital Combat Casualty Care: A Systematic Review. Prehosp Disaster Med 2020;35(6):676–682. PMID:32907690

134. Wu X, Zhang T, Chen L, Chen X. Prolonging the flush-lock interval of totally implantable venous access ports in patients with cancer: A systematic review and meta-analysis. J Vasc Access 2021;22(5):814–821. PMID:32873129

135. Ofstead CL, Buro BL, Hopkins KM, Eiland JE. The impact of continuous electrical microcurrent on acute and hard-to-heal wounds: A systematic review. J Wound Care 2020;29:S6–S15. PMID:32654615

136. Pinto-Fernandez D, Torricelli D, Sanchez-Villamanan MDC, Aller F, Mombaur K, Conti R, Vitiello N, Moreno JC, Pons JL. Performance Evaluation of Lower Limb Exoskeletons: A Systematic Review. IEEE Trans Neural Syst Rehabil Eng 2020;28(7):1573–1583. PMID:32634096

137. Park JH, Lee JH, Lee S, Shin JY, Kim TH. Adverse events related to electroacupuncture: a systematic review of single case studies and case series. Acupunct Med 2020;38(6):407–416. PMID:32418438

138. Liu Y, Sun JY, Zhu YS, Li ZM, Li KL, Wang RX. Association between CRT(D)/ICD and renal insufficiency: A systematic review and meta-analysis. Semin Dial 2021;34(1):17–30. PMID:33296540

139. Corrà MF, Warmerdam E, Vila-Chã N, Maetzler W, Maia L. Wearable health technology to quantify the functional impact of peripheral neuropathy on mobility in Parkinson’s disease: A systematic review. Sensors (Switzerland) 2020;20(22):1–34. PMID:33228056

140. Escamilla-Nunez R, Michelini A, Andrysek J. Biofeedback systems for gait rehabilitation of individuals with lower-limb amputation: A systematic review. Sensors (Switzerland) 2020;20(6). PMID:32183338

141. Ha L, Mizrahi D, Wakefield CE, Cohn RJ, Simar D, Signorelli C. The Use of Activity Trackers in Interventions for Childhood Cancer Patients and Survivors: A Systematic Review. J Adolesc Young Adult Oncol 2021;10(1):1–14. PMID:32897805

142. Düking P, Zinner C, Reed JL, Holmberg HC, Sperlich B. Predefined vs data-guided training prescription based on autonomic nervous system variation: A systematic review. Scand J Med Sci Sport 2020;30(12):2291–2304. PMID:32785959

143. Carreiro S, Newcomb M, Leach R, Ostrowski S, Boudreaux ED, Amante D. Current reporting of usability and impact of mHealth interventions for substance use disorder: A systematic review. Drug Alcohol Depend [Internet] Elsevier; 2020;215(August):108201. PMID:32777691

144. Wang SA, Su CP, Fan HY, Hou WH, Chen YC. Effects of real-time feedback on cardiopulmonary resuscitation quality on outcomes in adult patients with cardiac arrest: A systematic review and meta-analysis. Resuscitation [Internet] European Resuscitation Council, American Heart Association, Inc., and International Liaison Committee on Resuscitation.~Published by Elsevier Ireland Ltd; 2020;155(252):82–90. PMID:32755666

145. Nuss K, Moore K, Nelson T, Li K. Effects of Motivational Interviewing and Wearable Fitness Trackers on Motivation and Physical Activity: A Systematic Review. Am J Heal Promot 2020;1–10. [doi: 10.1177/0890117120939030]

146. Alghamdi A, Alotaibi A, Alharbi M, Reynard C, Body R. Diagnostic Performance of Prehospital Point-of-Care Troponin Tests to Rule out Acute Myocardial Infarction: A Systematic Review. Prehosp Disaster Med 2020;35(5):567–573. PMID:32641173

147. Asbeutah AAA, Salem MH, Asbeutah SA, Abu-Assi MA. The role of an antibiotic envelope in the prevention of major cardiac implantable electronic device infections: A systematic review and meta-analysis. Medicine (Baltimore) 2020;99(26):e20834. PMID:32590773

148. Ullah W, Nadeem N, Haq S, Thelmo FL, Abdullah HM, Haas DC. Efficacy of antibacterial envelope in prevention of cardiovascular implantable electronic device infections in high-risk patients: A systematic review and meta-analysis. Int J Cardiol [Internet] Elsevier B.V.; 2020;315(xxxx):51–56. PMID:32291170

149. Fawcett E, van Velthoven MH, Meinert E. Long-term weight management using wearable technology in overweight and obese adults: Systematic review. JMIR mHealth uHealth 2020;8(3):1–10. PMID:32154788

150. Husebo BS, Heintz HL, Berge LI, Owoyemi P, Rahman AT, Vahia I V. Sensing technology to facilitate behavioral and psychological symptoms and to monitor treatment response in people with dementia: A systematic review. Front Pharmacol 2020;10(February):1–13. [doi: 10.3389/fphar.2019.01699]

151. De Carvalho DE, de Luca K, Funabashi M, Breen A, Wong AYL, Johansson MS, Ferreira ML, Swab M, Neil Kawchuk G, Adams J, Hartvigsen J. Association of Exposures to Seated Postures With Immediate Increases in Back Pain: A Systematic Review of Studies With Objectively Measured Sitting Time. J Manipulative Physiol Ther [Internet] Elsevier Ltd; 2020;43(1):1–12. PMID:32081511

152. Pranata R, Tondas AE, Vania R, Yuniadi Y. Antibiotic envelope is associated with reduction in cardiac implantable electronic devices infections especially for high-power device—Systematic review and meta-analysis. J Arrhythmia 2020;36(1):166–173. [doi: 10.1002/joa3.12270]

153. Edwards SJ, Wakefield V, Jhita T, Kew K, Cain P, Marceniuk G. Implantable cardiac monitors to detect atrial fibrillation after cryptogenic stroke: A systematic review and economic evaluation. Health Technol Assess (Rockv) [Internet] 2020;24(5):v–184. PMID:31944175

154. McCarthy KJ, Locke AH, Coletti M, Young D, Merchant FM, Kramer DB. Outcomes following implantable cardioverter–defibrillator generator replacement in adults: A systematic review. Hear Rhythm [Internet] Elsevier Inc.; 2020;17(6):1036–1042. PMID:31931173

155. Wang E, Abrahamson K, Liu PJ, Ahmed A. Can Mobile Technology Improve Weight Loss in Overweight Adults? A Systematic Review. West J Nurs Res 2020;42(9):747–759. PMID:31762402

156. Barra S, Providência R, Narayanan K, Boveda S, Duehmke R, Garcia R, Leyva F, Roger V, Jouven X, Agarwal S, Levy WC, Marijon E. Time trends in sudden cardiac death risk in heart failure patients with cardiac resynchronization therapy: A systematic review. Eur Heart J 2020;41(21):1976–1986. PMID:31750896

157. Halawa A, Enezate T, Flaker G. Device monitoring in heart failure management: Outcomes based on a systematic review and meta-analysis. Cardiovasc Diagn Ther 2019;9(4):386–393. [doi: 10.21037/cdt.2019.01.02]

158. Albergoni A, Hettinga FJ, La Torre A, Bonato M, Sartor F. The Role of Technology in Adherence to Physical Activity Programs in Patients with Chronic Diseases Experiencing Fatigue: a Systematic Review. Sport Med - Open Sports Medicine - Open; 2019;5(1). [doi: 10.1186/s40798-019-0214-z]

159. Steinhaus DA, Lubitz SA, Noseworthy PA, Kramer DB. Exercise Interventions in Patients with Implantable Cardioverter-Defibrillators and Cardiac Resynchronization Therapy: A SYSTEMATIC REVIEW and META-ANALYSIS. J Cardiopulm Rehabil Prev 2019;39(5):308–317. PMID:31397767

160. Demiris G, Iribarren SJ, Sward K, Lee S, Yang R. Patient generated health data use in clinical practice: A systematic review. Nurs Outlook [Internet] Elsevier Inc.; 2019;67(4):311–330. PMID:31277895

161. Chen MY, Orkaby AR, Rosenberg MA, Driver JA. Frailty, Implantable Cardioverter Defibrillators, and Mortality: a Systematic Review. J Gen Intern Med Journal of General Internal Medicine; 2019;34(10):2224–2231. PMID:31264082

162. Dorri S, Asadi F, Olfatbakhsh A, Kazemi A. A Systematic Review of Electronic Health (eHealth) interventions to improve physical activity in patients with breast cancer. Breast Cancer [Internet] Springer Japan; 2020;27(1):25–46. PMID:31187411

163. Buckingham SA, Williams AJ, Morrissey K, Price L, Harrison J. Mobile health interventions to promote physical activity and reduce sedentary behaviour in the workplace: A systematic review. Digit Heal 2019;5:1–50. [doi: 10.1177/2055207619839883]

164. Rattanawong P, Kewcharoen J, Mekraksakit P, Mekritthikrai R, Prasitlumkum N, Vutthikraivit W, Putthapiban P, Dworkin J. Device infections in implantable cardioverter defibrillators versus permanent pacemakers: A systematic review and meta-analysis. J Cardiovasc Electrophysiol 2019;30(7):1053–1065. PMID:30938929

165. Alharbi M, Straiton N, Smith S, Neubeck L, Gallagher R. Data management and wearables in older adults: A systematic review. Maturitas [Internet] Elsevier; 2019;124(February):100–110. PMID:30910279

166. Masri A, Altibi AM, Erqou S, Zmaili MA, Saleh A, Al-Adham R, Ayoub K, Baghal M, Alkukhun L, Barakat AF, Jain S, Saba S, Adelstein E. Wearable Cardioverter-Defibrillator Therapy for the Prevention of Sudden Cardiac Death: A Systematic Review and Meta-Analysis. JACC Clin Electrophysiol 2019;5(2):152–161. PMID:30784684

167. Robbins R, Seixas A, Walton Masters L, Chanko N, Diaby F, Vieira D, Jean-Louis G. Sleep Tracking: a Systematic Review of the Research Using Commercially Available Technology. Curr Sleep Med Reports Current Sleep Medicine Reports; 2019;5(3):156–163. [doi: 10.1007/s40675-019-00150-1]

168. Naka KK, Bazoukis G, Bechlioulis A, Korantzopoulos P, Michalis LK, Ntzani EE. Association between atrial fibrillation and patient-important outcomes in heart failure patients with implantable cardioverter-defibrillators: A systematic review and meta-analysis. Eur Hear J - Qual Care Clin Outcomes 2019;5(2):96–104. PMID:30462233

169. Voruganti DC, Briasoulis A, Chaudhry M, Alvarez P, Cotarlan V, Bhama JK, Giudici M. Cardiac resynchronization therapy and outcomes in patients with left ventricular assist devices: a systematic review and meta-analysis. Heart Fail Rev Heart Failure Reviews; 2019;24(2):229–236. PMID:30259285
