# Supplementary material for: Digital Biomarker–Based Studies: Scoping Review of Systematic Reviews
Source: JMIR Mhealth Uhealth. 2022 Oct 24;10(10):e35722. doi: 10.2196/35722 (PMC9641516; doi:10.2196/35722)
Supplement: Multimedia Appendix 5 [file mhealth_v10i10e35722_app5.docx]

| References | Physiological/behavioral data | Digital devices | Type of digital devices | Role of digital biomarkers | Type of sensors |
| --- | --- | --- | --- | --- | --- |
| [1] | Heart functions, Gait pattern functions, Temperature | smart glasses, smart watches, smart bracelets, smart shoes, smart socks | wearable | intervention, measure of outcome, diagnostic tool | heart rate sensors/pulse rate sensors, temperature sensors, position sensors |
| [2] | Heart rate, Respiration functions, Quality of sleep, Maintenance of blood pressure, Looking after one's health | inertial measurement unit sensors, continuous glucose monitoring systems | wearable | measure of outcome | heart rate sensors/pulse rate sensors, chemical sensors |
| [3] | Running, Looking after one's health, Weight maintenance functions | Fitbit | wearable | intervention | position sensors |
| [4] | Heart functions | ICD ^a^, iPhone-based rhythm monitoring device, pacemakers | implantable | intervention | heart rate sensors/pulse rate sensors |
| [5] | Attitudes | Fitbit Zip, AiperMotion 500, LegSysTM, LUMO, Polar S410TM | wearable | intervention | other |
| [6] | Looking after one's health, Running, Walking | activity monitor, portable tablet computers with touch screens, Fitbit, Jawbone UP24 wearable device, pedometer, accelerometer | Wearable, portable | intervention | position sensors |
| [7] | Walking, Weight maintenance functions | Fitbit, Jawbone Up24, Combined HR monitor and accelerometer (Actiheart), Wrist-worn accelerometer, FIT Core, Body Media, Fitbug Orb, Polar FA20 accelerometer, | wearable | intervention | position sensors |
| [8] | Walking | Pedometer | wearable | intervention | position sensors |
| [9] | Looking after one's health, Walking | Accelerometer, pedometer | wearable | intervention | position sensors |
| [10] | Looking after one's health, Walking | pedometer | wearable | intervention | position sensors |
| [11] | Heart rhythm | implantable cardioverter defibrillator (ICD) | implantable | intervention | heart rate sensors/pulse rate sensors |
| [12] | Heart rhythm | implantable cardioverter defibrillator (ICD) | implantable | intervention | heart rate sensors/pulse rate sensors |
| [13] | Looking after one's health, Walking | Fitbit, Jawbone UP, Polar Active, Misfit Flash, Gruve Solution, LUMOback, BodyMedia Fit, SenseWear, ActiveLink, InBodyBand | wearable | intervention | position sensors |
| [14] | Heart rhythm | implantable cardioverter defibrillator (ICD) | implantable | other | heart rate sensors/pulse rate sensors |
| [15] | Looking after one's health, Weight maintenance functions, Blood pressure functions | pedometers, accelerometers | wearable | intervention | position sensors, heart rate sensors/pulse rate sensors |
| [16] | Heart rhythm | implantable cardioverter defibrillator (ICD) | implantable | intervention | heart rate sensors/pulse rate sensors |
| [17] | Walking, Aerobic capacity | Garmin, Pedometer, Fitbit, Accelerometer, Yamax Digiwalker, Gex sensor of vital signs and smartphone, | wearable | intervention | position sensors |
| [18] | Weight maintenance functions | wristbands, smartwatches | wearable | intervention | position sensors |
| [19] | Weight maintenance functions, Blood pressure functions | FitBit, wearable glucometer and an activity monitor | wearable | intervention | heart rate sensors/pulse rate sensors, position sensors |
| [20] | Heart rhythm | ICD, pacemaker | implantable | intervention | heart rate sensors/pulse rate sensors |
| [21] | Looking after one's health, Weight maintenance functions | fitbit, pedometer, Fitmeter | wearable | measure of outcome | position sensors |
| [22] | Heart rhythm | implantable cardioverter defibrillator (ICD) | implantable | other | heart rate sensors/pulse rate sensors |
| [23] | Walking, Looking after one's health | Fitbit, Jawbone UP24, Gruve, LumoBack, Polar Active, Fitbug, Pebble+, Fitmeter, Personal Activity Monitor, Withings Pulse | wearable | intervention | Position sensor |
| [24] | Looking after one's health, Weight maintenance functions | Gruve, Fitbit Zip, iWell OSN, Bluetooth Actiwatch, Jawbone Up24, PAM, Philips DirectLife, Polar FA20, SenseWear, BodyMedia Fit Core, BodyMedia FIT System | wearable | intervention | Position sensor |
| [25] | Looking after one's health, Walking | Accelerometer, Dynaport MoveMonitor, Pedometer, Yamax Digi-walker CW700, ActivPal, ActiGraph, Personal Activity Monitor | wearable | measure of outcome | Position sensor |
| [26] | Heart rhythm | implantable cardioverter defibrillator (ICD) | implantable | other | heart rate sensors/pulse rate sensors |
| [27] | Functions related to pregnancy | Fitbit | wearable | intervention | position sensors |
| [28] | Looking after one's health | Fitbit, Yorbody, AiperMotion | wearable | intervention | Position sensor |
| [29] | Looking after one's health | pedometer | wearable | intervention | Position sensor |
| [30] | Looking after one's health, Walking | pedometers, Wii Fit, heart rate monitor | wearable | intervention | Position sensor |
| [31] | Walking and moving | pedometer | wearable | intervention | position sensors |

### ^a^ICD: Implantable cardioverter defibrillator

References

1. Lu L, Zhang J, Xie Y, Gao F, Xu S, Wu X, Ye Z. Wearable health devices in health care: Narrative systematic review. JMIR mHealth uHealth 2020;8(11). PMID:33164904

2. Graña Possamai C, Ravaud P, Ghosn L, Tran VT. Use of wearable biometric monitoring devices to measure outcomes in randomized clinical trials: a methodological systematic review. BMC Med BMC Medicine; 2020;18(1):1–11. PMID:33153462

3. Ringeval M, Wagner G, Denford J, Paré G, Kitsiou S. Fitbit-Based Interventions for Healthy Lifestyle Outcomes: Systematic Review and Meta-Analysis. J Med Internet Res 2020;22(10). PMID:33044175

4. Jang JP, Lin HT, Chen YJ, Hsieh MH, Huang YC. Role of remote monitoring in detection of atrial arrhythmia, stroke reduction, and use of anticoagulation therapy - A systematic review and meta-analysis. Circ J 2020;84(11):1922–1930. PMID:33012748

5. Kamei T, Kanamori T, Yamamoto Y, Edirippulige S. The use of wearable devices in chronic disease management to enhance adherence and improve telehealth outcomes: A systematic review and meta-analysis. J Telemed Telecare 2020; [doi: 10.1177/1357633X20937573]

6. Liu JYW, Kor PPK, Chan CPY, Kwan RYC, Sze-Ki D. The effectiveness of a wearable activity tracker (WAT)-based intervention to improve physical activity levels in sedentary older adults: A systematic review and meta-analysis. Arch Gerontol Geriatr [Internet] Elsevier; 2020;91(March):104211. PMID:32739713

7. Tang MSS, Moore K, McGavigan A, Clark RA, Ganesan AN. Effectiveness of wearable trackers on physical activity in healthy adults: Systematic review and meta-analysis of randomized controlled trials. JMIR mHealth uHealth 2020;8(7):1–13. PMID:32706685

8. Asbeutah AAA, Salem MH, Asbeutah SA, Abu-Assi MA. The role of an antibiotic envelope in the prevention of major cardiac implantable electronic device infections: A systematic review and meta-analysis. Medicine (Baltimore) 2020;99(26):e20834. PMID:32590773

9. Kwan RYC, Salihu D, Lee PH, Tse M, Cheung DSK, Roopsawang I, Choi KS. El efecto de las intervenciones de e-salud que promueven la actividad física en las personas mayores. Eur Rev Aging Phys Act European Review of Aging and Physical Activity; 2020;17(1).

10. Burge AT, Cox NS, Abramson MJ, Holland AE. Interventions for promoting physical activity in people with chronic obstructive pulmonary disease (COPD). Cochrane Database Syst Rev 2020;2020(4). PMID:32297320

11. Gama F, Ferreira J, Carmo J, Costa FM, Carvalho S, Carmo P, Cavaco D, Morgado FB, Adragão P, Mendes M. Implantable cardioverter–defibrillators in trials of drug therapy for heart failure: A systematic review and meta-analysis. J Am Heart Assoc 2020;9(8). PMID:32290732

12. Alotaibi S, Hernandez-Montfort J, Ali OE, El-Chilali K, Perez BA. Remote monitoring of implantable cardiac devices in heart failure patients: a systematic review and meta-analysis of randomized controlled trials. Heart Fail Rev Heart Failure Reviews; 2020;25(3):469–479. PMID:32002732

13. Lynch C, Bird S, Lythgo N, Selva-Raj I. Changing the Physical Activity Behavior of Adults With Fitness Trackers: A Systematic Review and Meta-Analysis. Am J Heal Promot 2020;34(4):418–430. PMID:31858812

14. Kheiri B, Barbarawi M, Zayed Y, Hicks M, Osman M, Rashdan L, Kyi HH, Bachuwa G, Hassan M, Stecker EC, Nazer B, Bhatt DL. Antiarrhythmic Drugs or Catheter Ablation in the Management of Ventricular Tachyarrhythmias in Patients with Implantable Cardioverter-Defibrillators: A Systematic Review and Meta-Analysis of Randomized Controlled Trials. Circ Arrhythmia Electrophysiol 2019;12(11):1–10. PMID:31698933

15. Hodkinson A, Kontopantelis E, Adeniji C, Van Marwijk H, McMillan B, Bower P, Panagioti M. Accelerometer- and Pedometer-Based Physical Activity Interventions among Adults with Cardiometabolic Conditions: A Systematic Review and Meta-analysis. JAMA Netw Open 2019;2(10). PMID:31596494

16. Disertori M, Masè M, Rigoni M, Nollo G, Ravelli F. Declining clinical benefit of ICD in heart failure patients: Temporal trend of mortality outcomes from randomized controlled trials. J Cardiol [Internet] Japanese College of Cardiology; 2020;75(2):148–154. PMID:31526627

17. Hannan AL, Harders MP, Hing W, Climstein M, Coombes JS, Furness J. Impact of wearable physical activity monitoring devices with exercise prescription or advice in the maintenance phase of cardiac rehabilitation: Systematic review and meta-analysis. BMC Sports Sci Med Rehabil BMC Sports Science, Medicine and Rehabilitation; 2019;11(1):1–21. [doi: 10.1186/s13102-019-0126-8]

18. Yen HY, Chiu HL. The effectiveness of wearable technologies as physical activity interventions in weight control: A systematic review and meta-analysis of randomized controlled trials. Obes Rev 2019;20(10):1485–1493. PMID:31342646

19. Jo A, Coronel BD, Coakes CE, Mainous AG. Is There a Benefit to Patients Using Wearable Devices Such as Fitbit or Health Apps on Mobiles? A Systematic Review. Am J Med [Internet] Elsevier Inc.; 2019;132(12):1394-1400.e1. PMID:31302077

20. Tseng AS, Kunze KL, Lee JZ, Amin M, Neville MR, Almader-Douglas D, Killu AM, Madhavan M, Cha YM, Asirvatham SJ, Friedman PA, Gersh BJ, Mulpuru SK. Efficacy of Pharmacologic and Cardiac Implantable Electronic Device Therapies in Patients with Heart Failure and Reduced Ejection Fraction: A Systematic Review and Network Meta-Analysis. Circ Arrhythmia Electrophysiol 2019;12(6):1–13. PMID:31159582

21. Mitchell MS, Orstad SL, Biswas A, Oh PI, Jay M, Pakosh MT, Faulkner G. Financial incentives for physical activity in adults: Systematic review and meta-analysis. Br J Sports Med 2020;54(21):1259–1268. PMID:31092399

22. Martinez BK, Baker WL, Konopka A, Giannelli D, Coleman CI, Kluger J, Cronin EM. Systematic review and meta-analysis of catheter ablation of ventricular tachycardia in ischemic heart disease. Hear Rhythm [Internet] Elsevier Inc.; 2020;17(1):e206–e219. PMID:31082362

23. Brickwood KJ, Watson G, O’brien J, Williams AD. Consumer-based wearable activity trackers increase physical activity participation: Systematic review and meta-analysis. JMIR mHealth uHealth 2019;7(4). PMID:30977740

24. Sypes EE, Newton G, Lewis ZH. Investigating the use of an electronic activity monitor system as a component of physical activity and weight-loss interventions in nonclinical populations: A systematic review. J Phys Act Heal 2019;16(4):294–302. PMID:30849927

25. Braakhuis HEM, Berger MAM, Bussmann JBJ. Effectiveness of healthcare interventions using objective feedback on physical activity: A systematic review and metaanalysis. J Rehabil Med 2019;51(3):151–159. PMID:30843082

26. Km N, Ad Z, Rs T, Jh S, Lindschou J, Anderson L, Jc J, Sk B, Km N, Ad Z, Rs T, Jh S, Lindschou J, Anderson L, Jc J, Sk B. Nielsen KM, Zwisler AD, Taylor RS, Svendsen JH, Lindschou J, Anderson L, Jakobsen JC, Berg SK. 2019; [doi: 10.1002/14651858.CD011828.pub2.Copyright]

27. Chan KL, Chen M. Effects of social media and mobile health apps on pregnancy care: Meta-analysis. JMIR mHealth uHealth 2019;7(1):1–14. [doi: 10.2196/11836]

28. Kirk MA, Amiri M, Pirbaglou M, Ritvo P. Wearable Technology and Physical Activity Behavior Change in Adults With Chronic Cardiometabolic Disease: A Systematic Review and Meta-Analysis. Am J Heal Promot 2019;33(5):778–791. PMID:30586996

29. Freak-Poli RLA, Cumpston M, Albarqouni L, Clemes SA, Peeters A. Workplace pedometer interventions for increasing physical activity. Cochrane Database Syst Rev 2020;2020(7). PMID:32700325

30. Schaffer K, Panneerselvam N, Poh Loh K, Herrmann R, Kleckner IR, Dunne RF, Lin PJ, Heckler CE, Gerbino N, Bruckner LB, Storozynsky E, Ky B, Baran A, Mohile SG, Mustian KM, Fung C. Systematic review of randomized controlled trials of exercise interventions using digital activity trackers in patients with cancer. JNCCN J Natl Compr Cancer Netw 2019;17(1):57–63. PMID:30659130

31. Armstrong M, Winnard A, Chynkiamis N, Boyle S, Burtin C, Vogiatzis I. Use of pedometers as a tool to promote daily physical activity levels in patients with COPD: A systematic review and meta-analysis. Eur Respir Rev [Internet] 2019;28(154). PMID:31722891
