# Supplementary figures and images for: Digital Biomarker–Based Studies: Scoping Review of Systematic Reviews
Source: JMIR Mhealth Uhealth. 2022 Oct 24;10(10):e35722. doi: 10.2196/35722 (PMC9641516; doi:10.2196/35722)

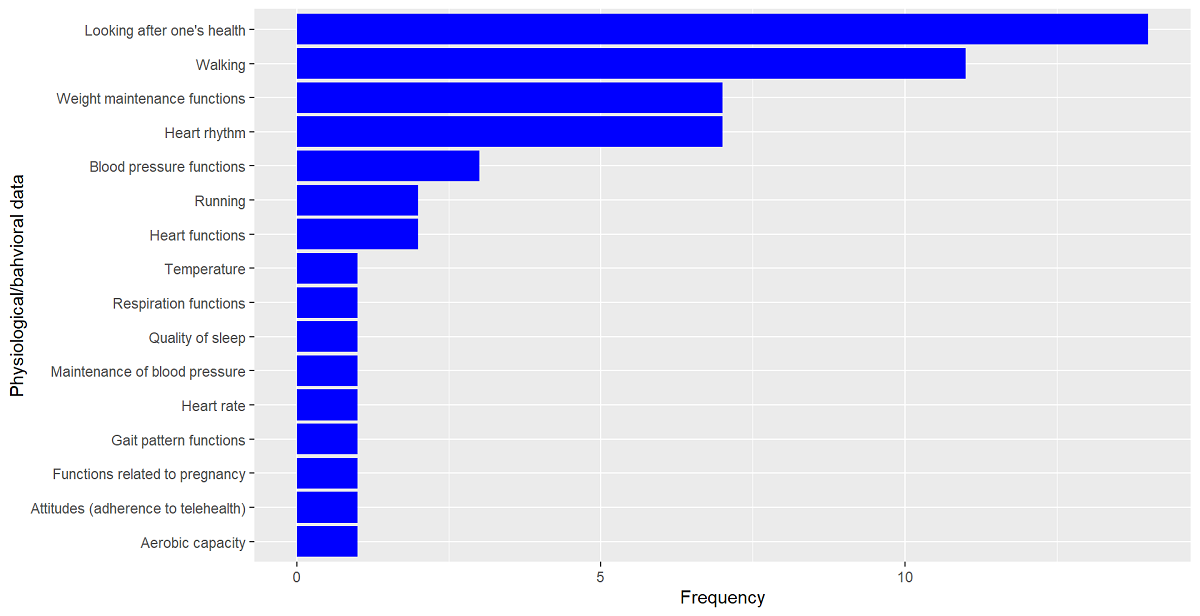

Supplement: Multimedia Appendix 6 [file mhealth_v10i10e35722_app6.png]
